# Bridging Scales in Alzheimer's Disease: Biological Framework for Brain Simulation With The Virtual Brain

**DOI:** 10.3389/fninf.2021.630172

**Published:** 2021-04-01

**Authors:** Leon Stefanovski, Jil Mona Meier, Roopa Kalsank Pai, Paul Triebkorn, Tristram Lett, Leon Martin, Konstantin Bülau, Martin Hofmann-Apitius, Ana Solodkin, Anthony Randal McIntosh, Petra Ritter

**Affiliations:** ^1^Berlin Institute of Health at Charité - Universitätsmedizin Berlin, Berlin, Germany; ^2^Charité - Universitätsmedizin Berlin, Corporate Member of Freie Universität Berlin and Humboldt-Universität zu Berlin, Department of Neurology with Experimental Neurology, Brain Simulation Section, Berlin, Germany; ^3^Bernstein Center for Computational Neuroscience Berlin, Berlin, Germany; ^4^Institut de Neurosciences des Systèmes, Aix Marseille Université, Marseille, France; ^5^Fraunhofer Institute for Algorithms and Scientific Computing SCAI, Sankt Augustin, Germany; ^6^Behavioral and Brain Sciences, University of Texas at Dallas, Dallas, TX, United States; ^7^Baycrest Health Sciences, Rotman Research Institute, Toronto, ON, Canada; ^8^Einstein Center for Neuroscience Berlin, Berlin, Germany; ^9^Einstein Center Digital Future, Berlin, Germany

**Keywords:** Alzheimer's disease, The Virtual Brain, brain simulation, multi-scale brain modeling, connectomics

## Abstract

Despite the acceleration of knowledge and data accumulation in neuroscience over the last years, the highly prevalent neurodegenerative disease of AD remains a growing problem. Alzheimer's Disease (AD) is the most common cause of dementia and represents the most prevalent neurodegenerative disease. For AD, disease-modifying treatments are presently lacking, and the understanding of disease mechanisms continues to be incomplete. In the present review, we discuss candidate contributing factors leading to AD, and evaluate novel computational brain simulation methods to further disentangle their potential roles. We first present an overview of existing computational models for AD that aim to provide a mechanistic understanding of the disease. Next, we outline the potential to link molecular aspects of neurodegeneration in AD with large-scale brain network modeling using The Virtual Brain (www.thevirtualbrain.org), an open-source, multiscale, whole-brain simulation neuroinformatics platform. Finally, we discuss how this methodological approach may contribute to the understanding, improved diagnostics, and treatment optimization of AD.

## Introduction

Every second senior with age above 90 years suffers from Alzheimer's disease (AD) or another dementia (Robinson et al., 2018a). The US's mortality rate for people with this neurodegenerative disease exceeds that of breast cancer and prostate cancer combined (Alzheimer's Association, 2019). Beyond the impact on patients' and their families' life circumstances, neurodegenerative diseases have an enormous economic impact and hence pose a massive societal burden. The Alzheimer's Association's latest report estimates the annual medical and care costs attributed to AD in the US at $290 billion in 2019 (Alzheimer's Association, 2019). By 2050, this number is expected to rise as high as $1.1 trillion (Alzheimer's Association, 2018). It is stated in the same report that early diagnosis at the stage of mild cognitive impairment (MCI) could save up to $7.9 trillion in cumulated medical and care costs by the year 2050. While the prevalence of AD, the most common type of neurodegenerative disease (Robinson et al., 2018a; Alzheimer's Association, 2019), increases, the underlying disease mechanisms are still not understood. No disease-modifying treatment exists for AD.

Despite the collection of large data sets and major advances in high throughput computational methods, theoretical frameworks that link the many pieces of observations together can aim to infer novel insights about the underlying causes (Ritter et al., 2013; Schirner et al., 2018; Solodkin et al., 2018; McIntosh and Jirsa, 2019). The brain has multiple organization levels (e.g., molecular, cellular, ensemble- and region-level), including both feedback and feedforward interactions between and within those different levels (Solodkin et al., 2018). These dependencies are non-linear, leading to emergent phenomena—features of the system that cannot be understood by the simple “sum” of its parts (Ritter et al., 2013). Small perturbations in such non-linear systems can have enormous, widespread consequences. In the brain, interactions traverse many spatial and temporal scales, thus focusing on one scale can underestimate the emergent phenomena at other scales. Integrative brain modeling allows for the analysis of these multiple scales in parallel (Schirner et al., 2018), while computational neuroscience provides mathematical tools as the analysis of structured flows on manifolds (McIntosh and Jirsa, 2019) to understand the underlying dynamics.

A mechanistic understanding of AD could open new horizons for early diagnostics and cause-targeting treatments. Recent pharmacological clinical trials testing substances such as anti-Amyloid-beta (Gilman et al., 2005; Lannfelt et al., 2008; Winblad et al., 2012; Farlow et al., 2015; Sevigny et al., 2016; Vandenberghe et al., 2017; Panza et al., 2019), tau-protein targeting (Yanamandra et al., 2015; Bachurin et al., 2017; Jadhav et al., 2019), and immune-modulating substances used for rheumatoid arthritis (Jaturapatporn et al., 2012; Chou et al., 2016) have experienced significant setbacks (Panza et al., 2019). The development of novel therapeutics would benefit from theoretical and computational approaches (Hofmann-Apitius et al., 2015; Selkoe and Hardy, 2016; Solodkin et al., 2018).

**Figure 1:**
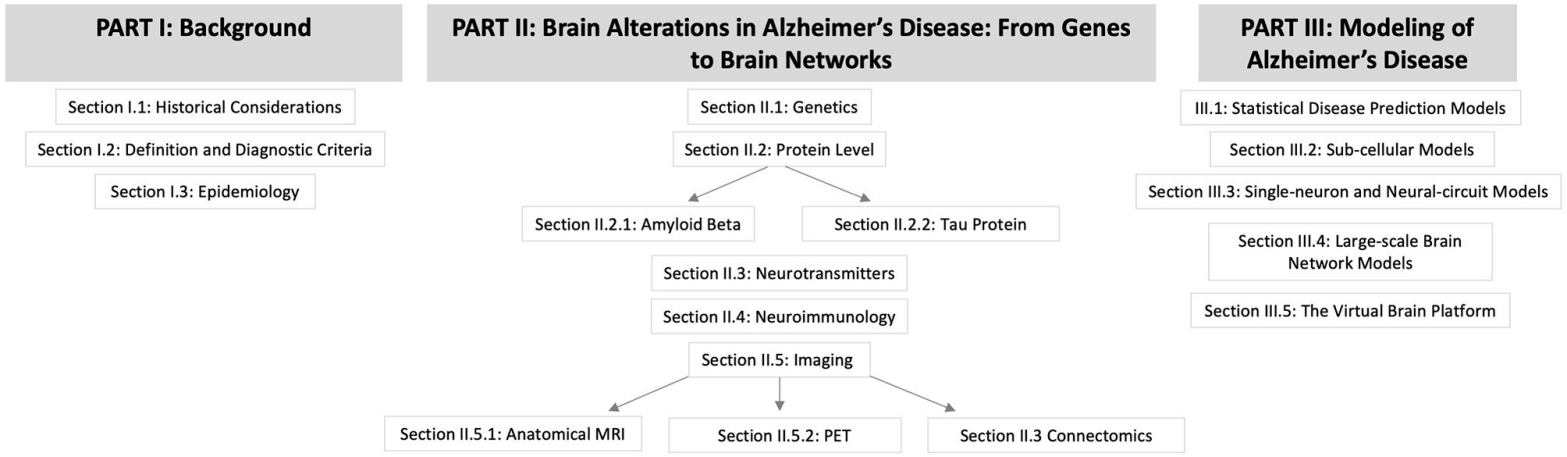
Flowchart for the structure of this article.

We hypothesize that an important contribution to understanding and curing AD lies in characterizing the features and processes that control emergent phenomena in the brain. A deep understanding of state-of-the-art biological research on AD and detailed knowledge of computational brain modeling tools are essential to reach this goal. In this review, we summarize current findings of AD pathogenesis from genomics to connectomics—describing the contribution of the classic hallmark proteins as well as current research on the Notch-1 pathway, neurotransmitters, polygenetic factors, neuroinflammation, and neuroplasticity. In the second part, we present various previous approaches to computational modeling of AD disease mechanisms and discuss their benefits and disadvantages. The last part describes The Virtual Brain (www.thevirtualbrain.org) as a multiscale brain simulation platform that enables linking molecular signaling cascades with large-scale brain simulation. An overview of the structure of this article is given by the flowchart in Figure 1.

## Background

### Historical Considerations

While dementia is nowadays a descriptive term for (acquired) severely impaired cognitive function due to a brain disease, dementia has historically been understood as a mainly physiological loss of mental function in the elderly (Schorer, 1985). Psychiatrists had therefore contrasted cognitive disorders of young people *(“dementia praecox”*), classified today as schizophrenia, for example, with dementia in old people, “*dementia senilis”*—i.e., the classification of dementia was dependent on the age at which the cognitive impairment occurred (Kendler, 2009). A profound challenge to this concept was posted in 1907 by an observation of Alois Alzheimer. His patient, Auguste D., had characteristic psychopathology of *dementia senilis* rapidly progressing—at an early age of 56 years (Alzheimer, 1907, 1911). Based on his observations of an “unusual disease,” a new neurologic and psychiatric research field emerged. Various mechanisms, risk factors, etiologic components (i.e., underlying causes such as neurotoxic proteins, risk-modifying genes, etc.), and comprehensive classifications of cognitive disorders were developed.

Interestingly much later in 2013 (Müller et al., 2013), it was shown that Auguste D. suffered from an early-onset variant of Alzheimer's dementia—a truly “unusual” and rare disease as one of the monogenetic forms with a mutation in the presenilin gene 1 (*PSEN1*) (Müller et al., 2013). Nevertheless, the primary cause of AD and also criteria for its diagnosis still remain unclear. Even the disease-defining biochemical findings of pathology in AD, represented by *Amyloid-beta* (Aβ40 and Aβ42, hereafter Abeta) and phosphorylation of *Tau protein* (TAU for *tubulin-associated unit* or by the Greek letter τ, hereafter Tau; for a review, see Bloom, 2014), remain controversial as causative of disease trajectory and cognitive symptoms. Nevertheless, their presence during pathogenesis is undisputable (Jellinger, 1997; Hyman et al., 2012; Nelson et al., 2012).

### Definition and Diagnostic Criteria

Nosology is the discipline of disease classification based on the underlying mechanisms. In this sense, a disease class can only be assigned if the respective disease's underlying etiology has been established. Otherwise, we speak of a “syndrome” or “disorder” (Jack et al., 2018). The dementia syndrome encompasses a broad array of different possible etiologies of cerebral or systemic origin (Wallesch and Förstl, 2012; Robinson et al., 2018a). Clinically it presents as a set of signs and symptoms. The affected neurocognitive domains are the higher cortical functions: memory, language, attentional processing, executive functions, and visuospatial domains. Different diagnostic criteria are currently used for the diagnosis of AD.

Among the most commonly used diagnostic criteria is the 1984 National Institute of Neurological and Communicative Disorders and Stroke and Alzheimer's Disease and Related Disorders Association (NINCDS-ADRDA) definition of possible and probable AD purely by clinical (e.g., daily-life impairments) and neuropsychological criteria without further diagnostic evaluation by technical means (McKhann et al., 1984). At the core of this definition are the presence and slow progression of cognitive decline in two or more cognitive domains, including memory, and the absence of alternative causes of dementia. The 2011 revision of these criteria further specified that impairment in activities of daily living is necessary for the diagnosis of dementia (McKhann et al., 2011).

In clinical practice, a diagnosis is mainly made based on the definition of probable AD according to NINCDS-ADRDA and by excluding possible other causes of dementia (Blennow et al., 2006). A challenge with this purely symptomatic definition is posed by the various phenomenological forms of AD (Wallesch and Förstl, 2012). Clinical symptoms and neurodegeneration occur on a continuum. They can vary tremendously between patients. The most common phenotype of AD is the slowly progressive amnestic variant. However, it is not uncommon for language disorders, disorientation, apraxia, or neuropsychiatric signs such as affective symptoms to appear first, while memory deficits do not seem to be predominant. This heterogeneity in phenotypes can have various causes, such as educational and social factors, the individual brain's structural vulnerability, or the patient's cognitive “reserve” (Stern, 2012).

The clinical definition of AD is further complicated by the overlap of symptoms with those of other dementias and comorbidities that can influence the clinical presentation. For example, if the patient also suffers from depression, possibly caused by beginning cognitive decline, this mood disorder itself can impact memory. And if patients also have Parkinson's disease—do they then necessarily have so-called Parkinson's dementia? Or do they suffer from AD and Parkinson's disease at the same time? Or has the neurodegeneration caused by Parkinson's disease diminished the cognitive reserve, which leads to the clinical onset of AD?

Unlike the original NINCDS-ADRDA classification, the current 2018 National Institute of Aging and Alzheimer's Association (NIA-AA) diagnostic definition is based on the presence of Abeta and Tau proteins in cerebrospinal fluid or positron emission tomography (PET) and atrophy indicating neurodegeneration in brain imaging. The NIA-AA definition introduces the so-called AT(N) classification to standardize biomarker findings in AD: therein A stands for positive Abeta biomarkers, T for phospho-Tau biomarkers, and N for Neurodegeneration markers in cerebrospinal fluid (total Tau burden) or atrophy shown in magnetic resonance imaging (MRI). Positive biomarkers are marked with a “+”-sign. As neurodegeneration is not specific for AD, N is usually placed in parentheses. The disease's cognitive dimension is defined separately and can be added to the classification as the letter C in its extension AT(N)(C). This definition is primarily intended for research and not used in routine clinical practice (Jack et al., 2018). It is debated whether this definition may prevent shifting scientific attention to other relevant candidate factors contributing to AD—which might lead to missing mechanistic cascades beyond Abeta and Tau proteins (Gauthier et al., 2018).

While the NINCDS-ADRDA definition only considers cognitive symptoms, the NIA-AA definition does not consider cognitive symptoms in their core AT(N) classification (Jack et al., 2018). Therefore, possible, more specific classifications could be “Alzheimer's disease with dementia” or “Alzheimer's disease with mild cognitive impairment” instead of the currently used term “Alzheimer's dementia,” which merges both AD pathologic changes and dementia syndromes (Jack et al., 2018).

Even though clinical classification for probable AD (McKhann et al., 1984) and research frameworks as the definition of AD by dementia with A+T+N+ biomarkers exist (Jack et al., 2018), only examination of invasively obtained tissue samples either from living individuals by biopsy or post-mortem at autopsy can provide a definitive diagnosis of AD—by proving the presence of neuritic plaques (with Abeta) or neurofibrillary tangles (with Tau). Autopsy is preferred to highly invasive *in vivo* interventions for a definitive diagnosis of AD, due to the lack of causal disease-modifying treatment options. The confirmation rate of clinically diagnosed AD by autopsy was calculated in a meta-analysis with a sensitivity of 85.4% and specificity of 77.7% (Cure et al., 2014). However, even neuropathological examination of brain tissue as the state-of-the-art gold standard method for AD diagnosis often reveals several protein-related pathologies, i.e., those representing the typical picture for AD plus others that have been associated with different neurodegenerative diseases (Robinson et al., 2018b).

Possible prodromal stages of dementia, e.g., mild cognitive impairment (MCI) and subjective cognitive decline, do not meet clinical criteria for a dementia syndrome because patients do not have deficits in their activities of daily living. In MCI, cognitive deficits are measurable but still do not affect activities of daily living (Petersen et al., 2014). In the case of subjective cognitive decline, it is not possible to objectively measure the deficits in neuropsychological examinations, but patients notice cognitive deficits themselves (Rabin et al., 2015). These states may in some cases be phase transitions of disease progression to dementia. In combination with other factors, they may help to assess an individual's risk of developing manifest dementia (Cheng et al., 2017).

Diagnosis is often an important question for patients and their families—even without helpful therapeutic interventions because it provides more certainty regarding the prognosis of the disease and the development of care plans. However, it is often unclear to what extent distinct types of dementia and related disorders contribute to cognitive impairment (Ashraf et al., 2016; Leyhe et al., 2017).

### Epidemiology

**Figure 2:**
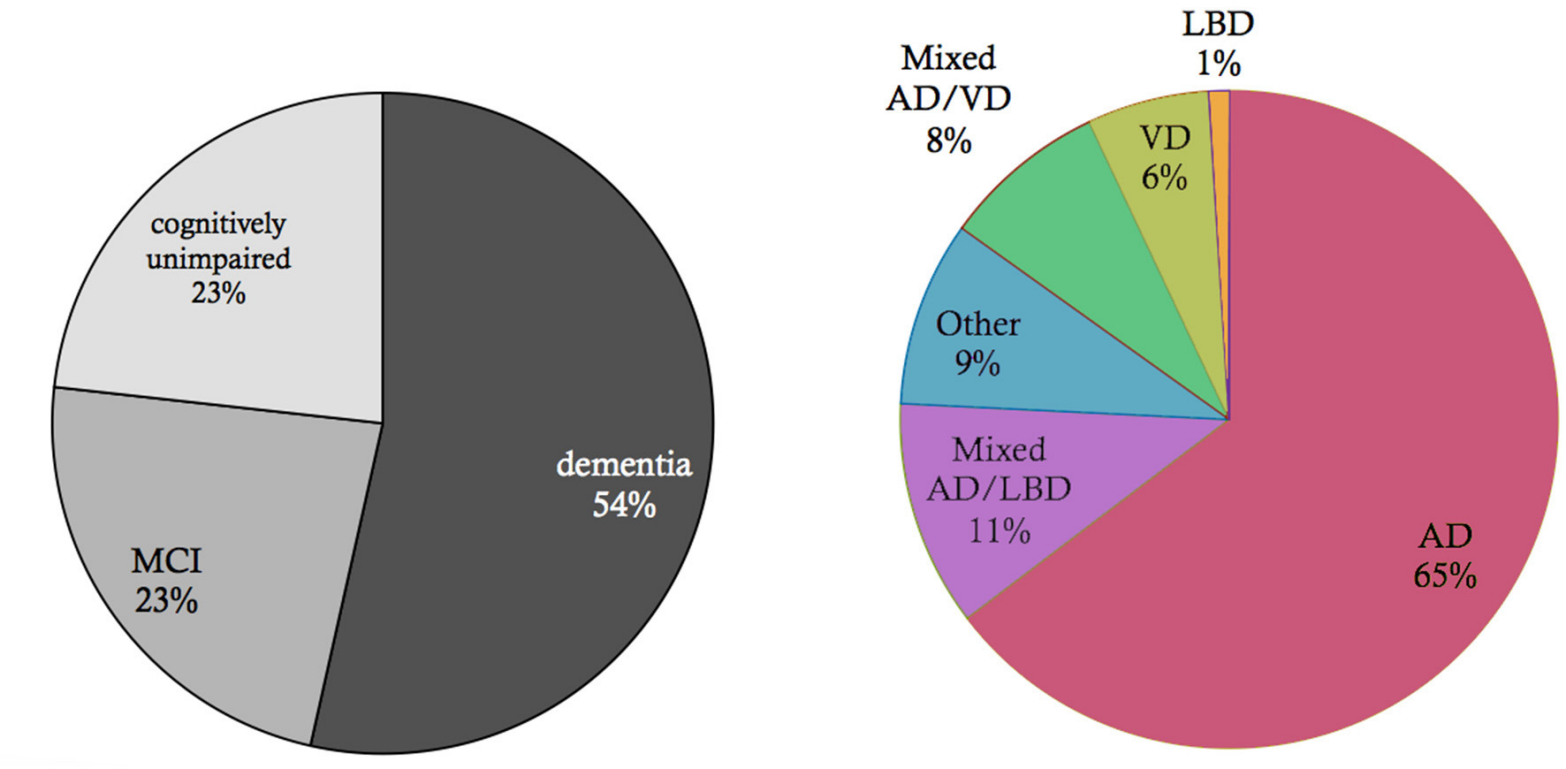
Basic epidemiology of different types of dementia. Data and *p*-values from Robinson et al. (2018a). Shown is an elderly cohort (*n* = 185) with a mean age of 97.7 years in an autopsy study. On the left, we see the prevalence of the cognitive states within this cohort at the time of death. More than half of the people suffered from dementia in this age group, while a quarter suffered from mild cognitive impairment (MCI), and another quarter had no cognitive disturbances. On the right, the clinical diagnosis (*ante mortem*) for the subpopulation that suffered from dementia is shown. Alzheimer's Disease (AD) is the most prevalent form of dementia; however, mixed forms and other primary neurodegenerative dementias as synucleinopathies or frontotemporal lobar degeneration (FTLD) spectrum also play a role as well as vascular dementia (VD). In the *post mortem* analysis, the full cohort showed at least partial AD-related pathologic changes: 100% had neurofibrillary tangles of at least Braak stage I, and 63% had neuritic plaques. The mean Braak stage was in the dementia group 4.1, in the non-dementia group 3.2 (*p* < 0.001). However, the dementia group also showed a significant higher Lewy-body pathology (*p* = 0.018) and transactive response DNA-binding protein 43 kDa (*TDP-43*) pathology (*p* < 0.001) as well as a higher rate of definitive cerebrovascular disease (*p* = 0.016). These findings indicate that in particular in the “super old,” different neuropathologic changes are probably concomitant and contribute to the development of cognitive decline in dementia—in contrast to the concept of “pure” AD as an isolated neurodegenerative disease.

**Figure 3:**
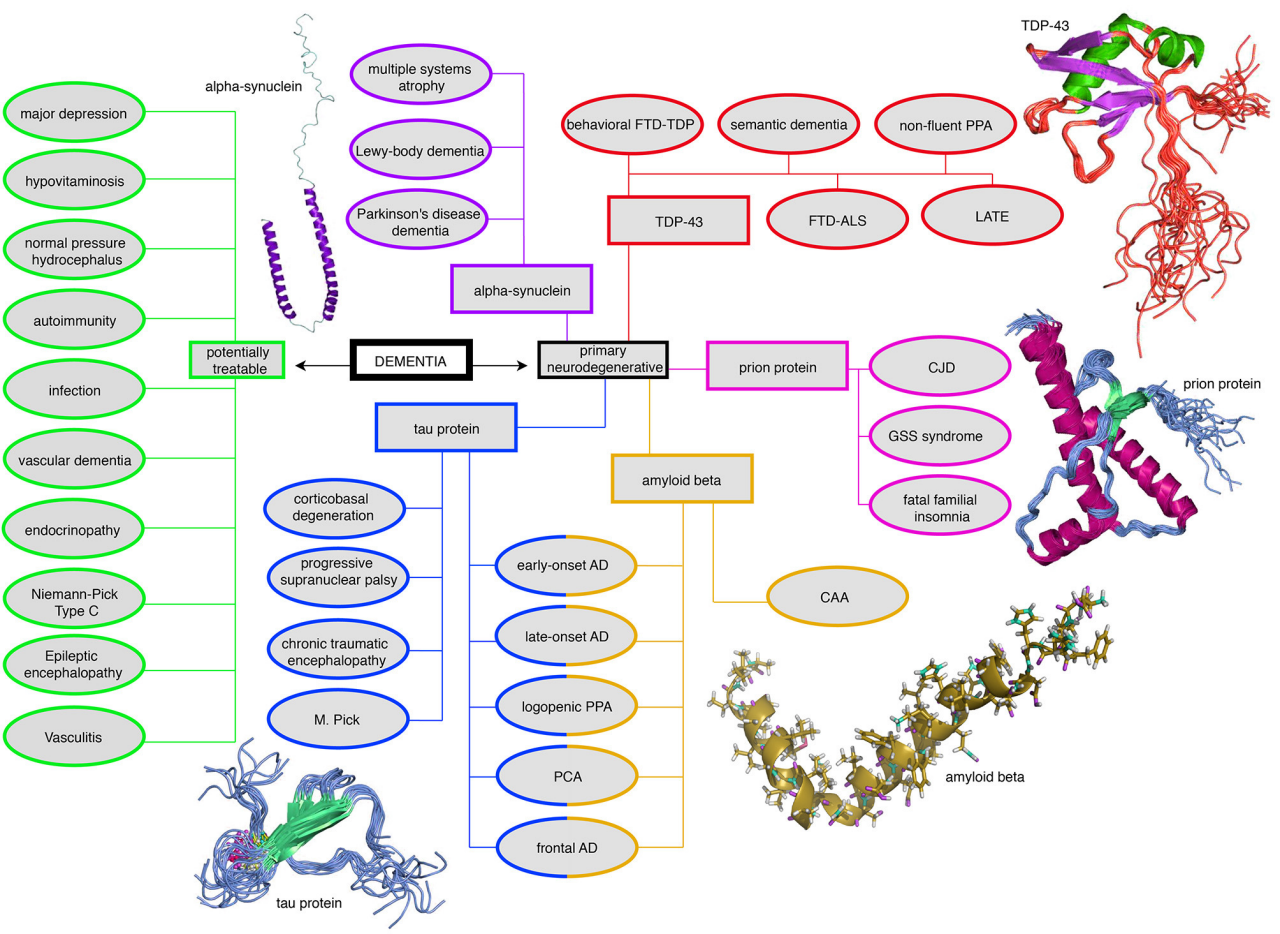
Mind map of the dementia syndrome and its differential diagnoses. The possible etiologies are widely spread across cerebral and systemic diseases. It is important to mention that Alzheimer's Disease (AD) is the most common form of dementia, but AD is not trivial to diagnose, in particular, if it requires to forgo some invasive tests in the elderly. However, the exact diagnosis is of enormous relevance because some possible causes of dementia are curable, such as normal pressure hydrocephalus, metabolic disorders, and immunologic or infectious causes. In the clinic, most patients are diagnosed with AD, vascular dementia, Lewy-body dementia, frontotemporal dementia, or a mixed form thereof (Figure 2). None of the primary neurodegenerative diseases can be treated in a causal and disease-modifying way, besides the treatment of vascular dementia with general atherosclerosis therapy. Their leading proteinopathy sorts the neurodegenerative diseases—caused by Abeta, Tau, prion protein, transactive response DNA binding protein 43 kDa (*TDP-43*), and alpha-synuclein (Wallesch and Förstl, 2012). Protein images modified from http://www.ebi.ac.uk/. FTD-TDP, frontotemporal degeneration caused by *TDP-43*; PPA, primary progressive aphasia; FTD-ALS, frontotemporal degeneration with amyotrophic lateral sclerosis; LATE, limbic-predominant age-related *TDP-43* encephalopathy; CJD, Creutzfeldt-Jakob's disease; GSS, Gerstmann-Sträußler-Scheinker (syndrome); CAA, cerebral amyloid angiopathy; PCA, posterior cortical atrophy; M. Pick, Pick's Disease.

Neurodegeneration is a continuum where several factors, such as proteinopathies, vascular and immunological changes, are likely to interact (Robinson et al., 2018b). Within the spectrum of dementias, the most common dementia is due to AD, followed by vascular dementia and mixed dementia which is a combination of AD and vascular dementia (American Psychiatric Association, 2013) (Figure 2, data from Robinson et al., 2018a). Frontotemporal dementias and Parkinsonian syndromes, particularly Lewy-body dementia and Parkinson's dementia, occur with lower prevalence than AD (Robinson et al., 2018a). A systematic overview of the most common and clinically important dementias is presented in Figure 3. Dementias can occur due to primary neurodegenerative diseases, i.e., AD, mixed dementia, frontotemporal and Lewy-body dementia, and due to secondary dementias linked to vascular changes, immunology, infections, and other diseases (Figure 3). Nevertheless, this differentiation is a simplification, as many secondary dementias arise from neurodegenerative processes during the disease course of primarily non-neurodegenerative diseases. A well-known example is the neurodegenerative course in late stages of multiple sclerosis (Bermel, 2017).

The relevance of a correct diagnosis of the specific form of dementia results from the different treatment strategies and prognoses related to different types of dementia. Some possible causes of dementia are curable, such as normal pressure hydrocephalus, metabolic disorders, and immunologic or infectious causes. While presently no disease-modifying therapy for any primary neurodegenerative disease exists (Alzheimer's Association, 2019), future therapies, as well as ongoing symptomatic and more experimental studies, may benefit from a careful patient stratification. This is particularly important for approaches that aim to model AD mechanisms with patient data, as the resulting model will only be as specific to AD as the patients' assigned correct (and strict) diagnosis. An overview of the experimental therapies that are in development for AD can be found in Table 1. For the established treatments for other dementia causes, we refer to Table 2.

**Table 1:** Ongoing developments for AD treatment and therapies.

**Possible disease-modifying treatment**	**Substance example**	**Results**	**References**
α secretase activators	Etazolate, Epigallocatechin gallate	Safety, Aβ aggregation↓	Vellas et al., 2011; Schneider et al., 2014
β secretase inhibitors	Pioglitazone, rosiglitazone, AZD3293	Safety, Plasma Aβ concentration↓cognitive benefit for diabetic patients in an observational study, until now no prospective clinical effect	Geldmacher et al., 2011; Read et al., 2014; Schneider et al., 2014; Heneka et al., 2015a,b; Cebers et al., 2017; Galimberti and Scarpini, 2017
γ secretase modulators	Tarenflurbil, *EVP-0962*	No clinical effect	Green et al., 2009; Morimoto, 2010; Schneider et al., 2014
γ secretase inhibitors	Semagacestat, Avagacestat	Skin cancer↑, infections↑, no clinical effect	Coric et al., 2012; Tong et al., 2012; Schneider et al., 2014
Aβ aggregation inhibitors	PBT2, Tramiprosat, Scylloinositol	CSF Aβ ↓, PiB PET↓, no clinical effect	Lannfelt et al., 2008; Faux et al., 2010; Schneider et al., 2014
Aβ active immunotherapy	Anti-Aβ vaccines AN1792, CAD-106	Meningoencephalitis (AN1792), positive antibody response, no clinical effect	Gilman et al., 2005; Winblad et al., 2012; Schneider et al., 2014; Farlow et al., 2015; Vandenberghe et al., 2017
Aβ passive immunotherapy	Solanezumab, Bapineuzumab	Safety, questionable cognitive effect of Solanezumab	Schneider et al., 2014; Siemers et al., 2016; Mo et al., 2017; Honig et al., 2018
τ phosphorylation inhibitors	Lithium, valproate	High toxicity, CSF τ ↓, and questionable cognitive effect of Lithium	Hampel et al., 2009; Forlenza et al., 2011; Schneider et al., 2014
τ fibrillization inhibitors	Methylene blue, davunetide	τ production ↓, possible cognitive effect of davunetide	Morimoto et al., 2013; Schneider et al., 2014
Macro-/Micronutrients	Polyunsaturated fatty acids	No clinical effect	Freund-Levi et al., 2006, 2009; Quinn et al., 2010; Schneider et al., 2014
Phosphodiesterase inhibitors	Cilostazol	Possible cognitive effect	Arai and Takahashi, 2009; Schneider et al., 2014
Tyrosine kinase inhibitors	Masitinib	unclear	Schneider et al., 2014; Folch et al., 2015
Statines	Simvastatin, atorvastatin	Unclear cognitive effects, CSF phopsho-τ ↓	Sano et al., 2011; Schneider et al., 2014; Li et al., 2017
Insulin	Intranasal insulin	FDG PET effect, possible cognitive effect	Craft et al., 2012; Schneider et al., 2014
NGF intracerebral application	Neurotrophic growth factor	CSF effects, gene expression effects, possible cognitive effect in the subgroup	Wahlberg et al., 2012; Tuszynski et al., 2015; Eyjolfsdottir et al., 2016
Deep brain stimulation	n.a.	Possible cognitive effects, highly invasive, ethical issues	Hardenacke et al., 2013; Salma et al., 2014; Nardone et al., 2015; Bittlinger and Muller, 2018; Lv et al., 2018
Transcranial brain stimulation	n.a.	Unclear effects	Freitas et al., 2011; Floel, 2014; Nardone et al., 2014, 2015; Rowan et al., 2014; Lefaucheur et al., 2017

*Parts of the table are modified from Schneider et al. (2014), Oertel (2017), and Young et al. (2018). Aβ, amyloid beta protein; CSF, cerebrospinal fluid; PiB PET/FDG PET, Pittsburgh Compound B/fluordeoxyglucose positron emission tomography; τ, tau protein*.

**Table 2:** Potentially curable causes of dementia syndromes (Wallesch and Förstl, 2012; Day, 2019).

**Curable dementia cause**	**Diagnostic tool**	**Therapy**
Major depression	Clinical	Psychotherapy, anti-depressive pharmacotherapy
Nutritive deficiency (Vitamine B12, D, folic acid)	Blood	Substitution
Infections (lues, borreliosis, viral)	CSF, Blood, clinical, imaging	Anti-infectious
Normal-pressure hydrocephalus	Imaging, tab test	Ventriculoperitoneal shunt
Autoimmune encephalitis	CSF, imaging	Immunosuppression, plasmapheresis
Vasculitis	CSF, imaging, angiography	Immunosuppression
Macroangiopathy	Imaging	Risk factor management, thrombendarteriectomy
Microangiopathy	Imaging	Risk factor management
Hypothyreosis	Blood	Substitution
Niemann-Pick type C	Blood (genetics, oxysterols)	Enzyme substitution
Epileptic encephalopathy	EEG, ex juvantibus	Anticonvulsive drugs

*CSF, cerebrospinal fluid; EEG, electroencephalography*.

The population over 80 years of age is the group in which the prevalence of dementia is increasing most rapidly (Fiest et al., 2016). While different pathogenic pathways have been hypothesized for AD and vascular dementia, it is increasingly acknowledged that both diseases share many risk factors (Love and Miners, 2016). However, interactions of Abeta in AD with vascular factors [e.g., altered blood-brain barrier permeability caused both by microvascular changes and Abeta deposition (Santos et al., 2017)] can be differentiated from cerebral amyloid angiopathy (Banerjee et al., 2020), a distinct vascular disease caused by amyloid, which we will not discuss further here.

An increased rate of cerebrovascular disease manifestations and thus a higher incidence of vascular dementia correlates with lifestyle and atherogenic risk factors such as physical activity (Lindsay et al., 2002; Larson et al., 2006), diabetes mellitus (Pasquier et al., 2006), and hypercholesterinemia (Shepardson et al., 2011a,b), which are also risk factors for AD (Reitz et al., 2011; Love and Miners, 2016). However, mechanisms whereby these factors mediate their impact, have been debated (Santos et al., 2017). One hypothesis is that microvascular lesions remain undiscovered, leading to a failure to diagnose vascular or mixed dementia. Another possibility is the involvement of metabolic pathways in the pathogenesis. Notably, the most important genetic risk factor in the general population is apolipoprotein E (*APOE*) E4 hetero- or monozygotic, an allele of a metabolic gene that also modulates atherosclerotic risk (Suri et al., 2013; Mahley, 2016). Especially in the elderly, the prevalence of both atherosclerosis and neurodegeneration increases exponentially and could likely affect the same individuals (Rohn, 2014). Although the role of metabolic factors is not clear, epidemiological approaches have shown that up to one-third of cases attributed to AD might be preventable by addressing these modifiable risk factors (Norton et al., 2014). However, this evidence comes from a mere observational method using the population-attributable risk. This statistical method describes the fraction of the incidence of a disease attributed to one particular risk factor. While this index allows an estimation of the effect that might follow removing the risk factor, as a result of an observational study, it precludes establishing a clear causal relation between observed risk factors and the disease (Siegerink and Rohmann, 2018). For example, if the definition of AD in the underlying observational study is inconsistent and therefore contains also other disease entities as mixed dementia, the population-attributable risk would be related to mixed dementia as well as to AD itself. Therefore, reducing atherosclerotic risk factors might affect the mixed dementia patients instead of “pure” AD cases.

## Brain Alterations in Alzheimer's Disease: From Genes to Brain Networks

### Genetics

Early-onset AD can be a familial disease with rare structural variants or copy number variants in genes that regulate Abeta production and clearance. For example, structural variants in the Amyloid beta precursor protein gene (*APP*) affect post-translational processing of APP by secretases leading to excess Abeta in early-onset AD. The presenilin 1 (*PSEN1*) and presenilin 2 (*PSEN2*) genes form the active component of the γ-secretase complex. It is critical for processing APP and other type-I integral membrane proteins, including members of the Notch signaling pathway and receptor tyrosine-protein kinase erbB-4 (*ERBB4*) (Sannerud et al., 2016). Autosomal dominant mutations of *PSEN1* and *PSEN2* affect endopeptidase and carboxypeptidase activity, leading to longer and more toxic forms of Abeta peptides (Ertekin-Taner, 2007; Lanoiselée et al., 2017). Other environmental and genetic factors may contribute to the etiology of early-onset AD (Sun et al., 2017).

In contrast, late-onset AD is a complex genetic disease in which rare structural variants and common variants, mostly identified by genome-wide association studies, play an influential role in etiology. The heritability of late-onset AD is estimated to be high, with ~50% (Pedersen et al., 2004; Ridge et al., 2016)—yet environmental factors are likely to be additionally important (Grant et al., 2002; Wainaina et al., 2014). The single nucleotide polymorphism based heritability estimates are usually high in AD, at ~25–30% (Cuyvers and Sleegers, 2016), compared to other complex genetic brain disorders (Speed et al., 2017; Visscher et al., 2017). *APOE* E2/E4 polymorphisms alone explain ~25% of the single nucleotide polymorphism-based heritability, while common single nucleotide polymorphisms explain the remaining 5–7% (Cuyvers and Sleegers, 2016; Ridge et al., 2016; Kunkle et al., 2019). The most recent three genome-wide association studies have identified 40 independent risk loci (Marioni et al., 2018; Jansen et al., 2019; Kunkle et al., 2019). The majority of these loci have functions in three main pathways: lipid metabolism, microglial activation, and APP processing (Andrews et al., 2020). Notably, many of these loci contain functionally relevant single nucleotide polymorphisms that impact expression in AD-associated cortical tissues and correlate with the so-called expression of quantitative trait loci (Kunkle et al., 2019).

### Protein Level

Two of the most relevant proteins associated with the pathogenesis of AD are Abeta and phosphorylated Tau. Abeta is a human protein that—due to an abnormally cleaved configuration—aggregates in neuritic plaques leading to its (neuro-)toxic effects (Klunk et al., 2007; Jack et al., 2009; Villemagne et al., 2009). It can be found both intra- and extracellularly (Hardy and Selkoe, 2002; Walsh and Selkoe, 2007; Selkoe and Hardy, 2016). It has been suggested that the hyperphosphorylation of Tau protein develops as a consequence of the aggregation of Abeta (Blennow et al., 2006). However, phosphorylated Tau is also present in other neurodegenerative diseases not associated with Abeta deposition (Kovacs, 2015). Immunohistochemical analysis of brain tissue, which is more sensitive than standard microscopical tissue examination, reveals up to 92–100% of Tau in people who died with neurodegenerative disorders at a mean age of 71 years, in contrast to Abeta with 20–57% (Robinson et al., 2018b). All subjects who met the official clinicopathological criteria for AD [ADNPC, defined as the presence of Abeta plaques, neurofibrillary tangles, and neuritic plaques (Montine et al., 2012)] in standard microscopy also showed Abeta and Tau in immunohistochemistry (Robinson et al., 2018b). However, in the same group of patients, two other protein pathologies were measured: alpha-synuclein (*SNCA*, associated with several diseases of the Parkinson spectrum) was present in 41–55% and transactive response DNA-binding protein 43 kDa (*TDP-43*, associated with amyotrophic lateral sclerosis and frontotemporal dementia) in 33–40% of patients (Figure 3) (Robinson et al., 2018b). Therefore, “pure” AD was a rare case in this cohort as 65–70% of confirmed AD patients suffered at least from one other neurodegenerative comorbidity (Robinson et al., 2018b).

#### Amyloid-Beta

**Figure 4:**
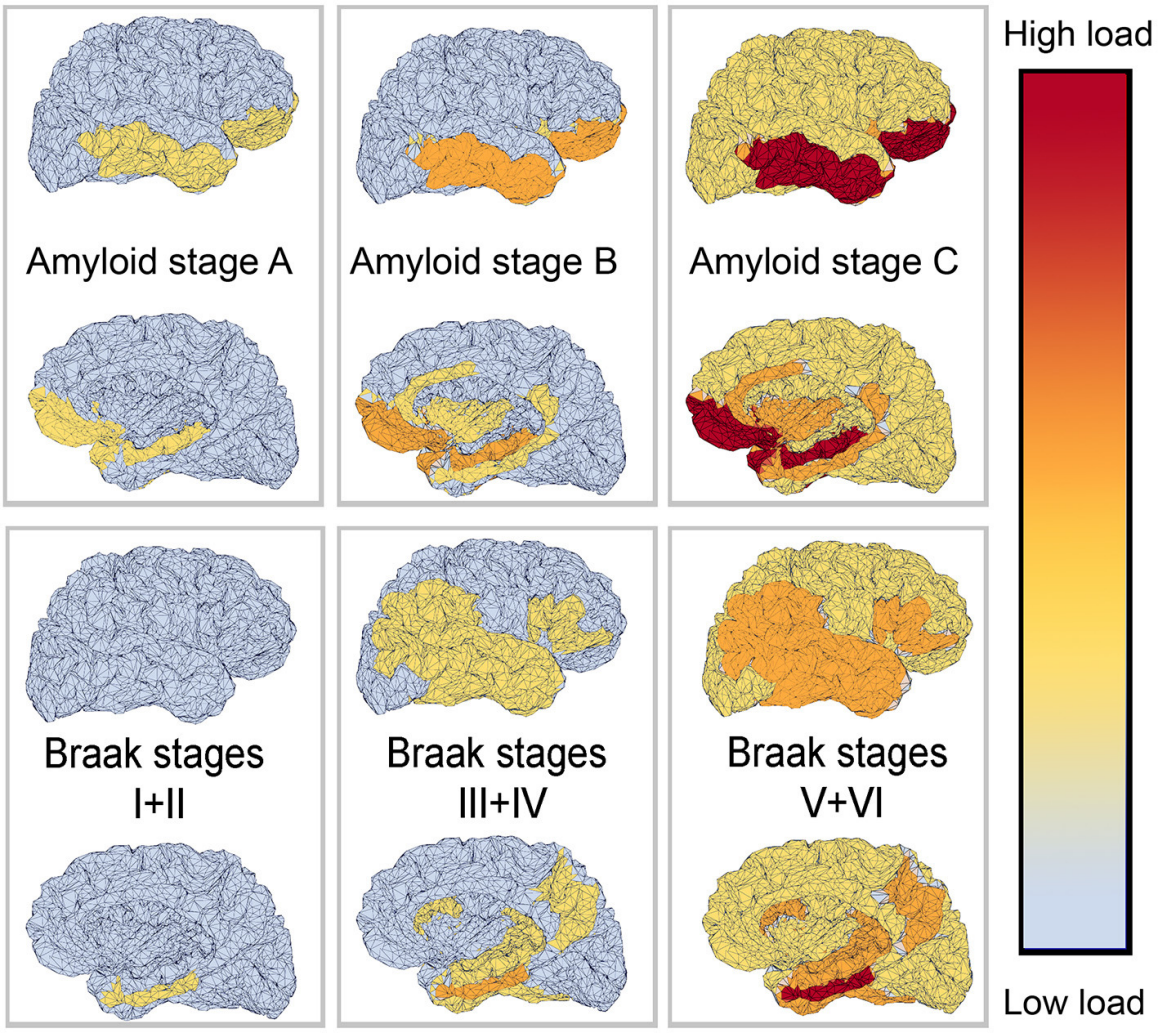
Visual representation of Abeta and Tau stages according to Braak and Braak (1991, 1997), and Braak et al. (2006). The darker color indicates a higher load of this protein in the respective brain area. The regions are listed in Tables 3, 4.

The deposition of Abeta typically follows a particular spatiotemporal pattern in the progression of AD. An illustration of this distribution, initially described by Braak and Braak (1991), can be seen in Figure 4. The course can be divided into three general stages (Braak and Braak, 1991, 1997; Taylor and Probst, 2008). Stage A develops along the perirhinal and entorhinal cortices. Stage B involves the hippocampus proper and neighboring regions like the posterior gyrus parahippocampalis. Stage C also encompasses a wide distribution of neocortical areas. Table 3 lists the brain regions that are specific to each amyloid stage.

**Table 3:** Stages of amyloid deposition (Braak and Braak, 1991, 1997; Braak et al., 2006).

**Amyloid deposition—**		
**Stage A**	**Stage B**	**Stage C**
Polar and orbitofrontal prefrontal cortex; polar, inferior, central, and ventral temporal cortex	As stage A, additional: hippocampus and gyrus parahippocampalis, amygdala, posterior and anterior insula, subgenual and retrosplenial cingulum, ventrolateral prefrontal cortex	As stage B, additional global neocortical dissemination

**Figure 5:**
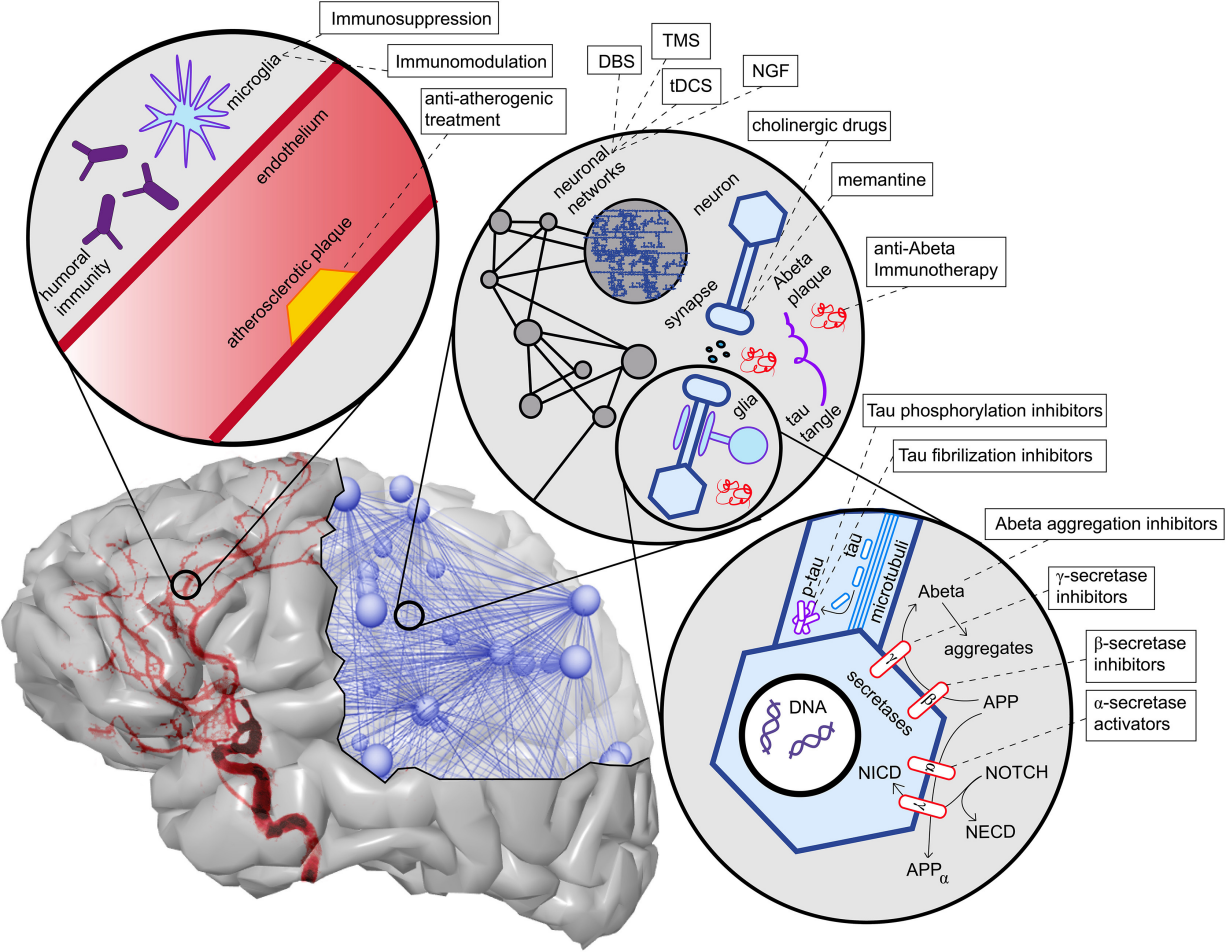
Overview of contributing factors in AD and potential intervention strategies. Shown are only the most important factors, which are also described in more detail in this article's main text. In the upper left corner, we see the neurovascular system. Both characteristics of blood vessels (e.g., atherosclerosis and endothelial dysfunction) (Love and Miners, 2016), as well as aspects of the blood-brain barrier (Sweeney et al., 2018), play a role in AD. A particular aspect here is the role of neural immunity, both with the brain-own microglia cells and the effect of systemic immune cells, e.g., mediated by antibodies (Heneka et al., 2015a,c). On the upper right corner, we see an illustration of the multiscale network structure of the brain. Stimulation approaches as deep brain stimulation (DBS), transcranial magnetic stimulation (TMS), and transcranial direct current stimulation (tDCS) act on the larger scale of a network-level; nevertheless, the actual changes happen on the level of synapses. Also, transmitter interventions develop their effects mainly at the micro-scale of synapses. In the lower right corner, basic molecular pathways in the extra- and intracellular space of a neuron are shown. We focused here on the processing of the two hallmark proteins Abeta and Tau, as well as the Notch-1 pathway, which is involved in memory (Marathe and Alberi, 2015) and plasticity (Brai et al., 2015). We illustrate the APP procession by the amyloidogenic or non-amyloidogenic way and its interaction with Notch-1 processing and, second, in the axon, the hyperphosphorylation and aggregation of Tau. A more detailed description of the named treatment strategies presently under development is provided in Table 1. NGF, nerve growth factor; Abeta, amyloid-beta; p-tau, phosphorylized Tau protein; APP, amyloid precursor protein; APPα, APP in alpha-helix configuration; NECD, Notch extracellular domain; NICD, Notch intracellular domain.

One possible explanation of the pathogenic deposition of Abeta is a maladaptive change in its processing, regulated by a group of secretases and other enzymes. We give only a brief overview here. APP is a transmembrane protein whose function has been associated with neural development and synaptic plasticity (Korte, 2019). It can be processed into different subdomains. One possible way is the subsequent procession by the α-secretase and the γ-secretase, called the non-amyloidogenic pathway (Blennow et al., 2006). This “physiological” pathway does not lead to Abeta fragments (with β-helix), which can later aggregate to plaques, but APP is transformed into a protein subdomain with α-helix configuration (Blennow et al., 2006). In another “pathological” pathway, APP is processed to soluble Abeta with a β-helix configuration by the β-secretase (and afterwards again by the γ-secretase). The β-helices allow molecules to aggregate into Abeta oligomers and afterwards polymers which become insoluble and deposit in the extracellular space, forming the so-called Abeta plaques. This pathway's activation leads to decreased Abeta concentration in cerebrospinal fluid because its insoluble configuration cannot be measured therein (Blennow et al., 2006; Olsson et al., 2016). The imbalance between these two pathways, represented by the activity of α- and the β-secretase, is suggested to play a major role in the pathogenesis of AD and is currently the target of various experimental treatment strategies (Coric et al., 2012; Tong et al., 2012; Ortega et al., 2013; Hsiao et al., 2019; Xia, 2019) (Table 1 and Figure 5).

Another important feature, linked not only to the protein metabolism of Abeta but also to neural development, is the notch receptor 1 (*NOTCH1*) pathway (Pierfelice et al., 2011; Brai et al., 2015; Marathe and Alberi, 2015). NOTCH1 is a membrane protein that plays a major role as the transcription factor for both its intracellular- and extracellular domain (Brai et al., 2016). The processing of NOTCH1 to its subdomains is performed by the γ-secretase—the same secretase involved in the amyloidogenic and non-amyloidogenic pathway of APP processing (Brai et al., 2016). APP and NOTCH1 are co-substrates in the extracellular domain of the γ-secretase (Marathe and Alberi, 2015; Brai et al., 2016). NOTCH1 is found in Abeta plaques, and its intracellular signaling is reduced in AD (Brai et al., 2016).

#### Tau Protein

In the absence of any additional neuropathological factors in several dementias, phosphorylated Tau may play a major role in the degenerative process of so-called primary tauopathies. This group includes, e.g., progressive supranuclear palsy (Cope et al., 2018), corticobasal degeneration, and the spectrum of frontotemporal dementia (including Pick's disease) (Kovacs, 2015). In contrast, in secondary tauopathies, Tau seems to be involved in the pathogenesis only when other factors are present, as, e.g., in prion diseases and chronic traumatic encephalopathy (Kovacs, 2015) (Figure 3). From this point of view, AD has a unique context because it is neither classified as a primary tauopathy (due to the concomitant presence of Abeta) nor is the amyloid pathology ultimately linked to Tau's hyperphosphorylation. However, phosphorylated Tau density correlates better with the severity of cognitive decline than the accumulation of Abeta itself (Riley et al., 2002; Bennett et al., 2005). Hence, there is controversy about the role of Tau protein in AD as either an independent disease factor or an indicator of general neurodegeneration derived from the neurotoxic effects of amyloid deposition. Clinical trials, based on anti-Tau-antibodies and -vaccines, modulators of Tau aggregation, and antisense oligonucleotides targeting its gene the microtubule associated protein tau (*MAPT*), are currently ongoing for AD patients (Yanamandra et al., 2015; Jadhav et al., 2019) (Table 1).

Tau protein pathology is complex and involved in several neurodegenerative processes (Kovacs, 2015; Guo et al., 2017; Cope et al., 2018). Different forms of neurodegeneration lead to the deposition of Tau (Spires-Jones et al., 2017), which can be measured in the cerebrospinal fluid (Ossenkoppele et al., 2015). In general, the phosphorylation homeostasis of the Tau protein is maintained by a series of kinases. A turn of this equilibrium toward hyperphosphorylation of Tau protein shows two consequential effects: first, Tau loses its natural function of microtubule stabilization followed by disturbed axonal transportation of vesicles, leading to disturbed axonal signal transmission. Second, the hyperphosphorylated Tau protein polymerizes to insoluble filaments and big tubular aggregates, the so-called neurofibrillary tangles. The brain's clearance system is unable to eliminate these aggregates leading to inflammatory processes and, eventually, neuronal death (Blennow et al., 2006). These phenomena have been observed using three methods: (i) microscopy of neuronal tissue with neurofibrillary tangles, (ii) an increased concentration of the hyperphosphorylated Tau section in the cerebrospinal fluid, and (iii) non-invasive nuclear imaging methods that trace Tau protein (flortaucipir PET) (Cope et al., 2018). Because of its two main effects, namely neuronal death and axonal dysfunction, Tau leads to a disconnection of the affected regions in the brain network. This has been measured in regions with high binding of flortaucipir PET tracing the Tau protein (Cope et al., 2018). However, the Tau protein is a better marker in diagnostics for the severity of cognitive dysfunction than Abeta in AD (Degerman Gunnarsson et al., 2014). The local neurotoxic effects of Tau can be linked to network disruption and an increased clinical score of apathy symptoms (Kitamura et al., 2018).

The stages of Tau deposition (defined by *post mortem* histopathological criteria), similar to Abeta stages, are called Braak Tau deposition stages (Braak and Braak, 1991, 1997; Braak et al., 2006), and show a characteristic spatiotemporal pattern formation in the course of typical AD. The patterns of *post mortem* neuropathological and nuclear imaging findings are illustrated in Table 4. For this reason, most patients with “typical” AD show early Tau depositions years before the onset of symptoms in the medial temporal lobe. The so-called transentorhinal stage consists of stages I and II and concerns the transentorhinal cortex in the ventromedial temporal lobe, and later the entorhinal cortex in the lamina granularis externa (Lamina II). This prodromal stage is followed by a further spreading into the limbic lobule (stages III and IV, involving mainly the hippocampus and the temporal allocortex) and finally into the neocortex (stages V and VI) (Braak and Braak, 1991, 1997; Braak et al., 2006). The six stages of this dissemination process of Tau deposition fall into three functional stages (i.e., transentorhinal/entorhinal, limbic, and neocortical), and have a high correlation to the cognitive decline of an individual AD patient (Riley et al., 2002; Bennett et al., 2005). Only a few amyloid plaques and often no clinical symptoms can be observed in this first functional category. In detail, the non-obligatory prodromal stage of AD is characterized by an MCI, which often converts to the full clinical presentation of dementia and has, thus, a significant correlation to higher Braak Tau deposition stages (Riley et al., 2002; Bennett et al., 2005). In the limbic stage, the Tau deposition is strongly associated with clinical symptoms of MCI stage (e.g., memory function, verbal fluency, impairments of daily life activities) (Riley et al., 2002). In the highest functional stage, which concerns the neocortex, most patients have amnestic impairment (Braak and Braak, 1991, 1997; Taylor and Probst, 2008). Similarly, the Tau protein deposition traced by flortaucipir PET correlates with the clinical presence of MCI as well as with AD and with cognitive performance (Cho et al., 2016).

**Table 4:** Braak stages of Tau deposition (Braak and Braak, 1991, 1997; Taylor and Probst, 2008).

**Tau deposition stage**		**Anatomic region**	**^**18**^F-AV-1451 PET**	**Diagnosis (PET)**
I	Trans-entorhinal	Transentorhinal cortex, perirhinal cortex (medial temporal lobe)	Entorhinal cortex, Hippocampus, Parahippocampal cortex (Cho et al., 2016)	MCI
II		Entorhinal cortex (lamina II)		
III	limbic	Hippocampus, temporal allocortex		
IV		Neocortical association fields next to the hippocampus	Isocortical spreading, particular differences to MCI in the precuneus, prefrontal, temporal, and inferior parietal cortex (Cho et al., 2016)	AD
V	Isocortical	Neocortex, spreading to dorsolateral		
VI		Primary sensory and motor areas		

*^18^F-AV-1451 PET, ^18^F-flortaucipir positron emission tomography; MCI, mild cognitive impairment*.

There seems to be a high correlation in general of Tau and amyloid deposition patterns described above; however, it is worth mentioning that the three stages of amyloid deposition described by Braak and Braak (Braak and Braak, 1991, 1997; Taylor and Probst, 2008), A, B, and C, do not strictly coincide with the Tau deposition stages I–VI. The six stages of Tau deposition follow a stricter distribution course and show some overlap with the amyloid deposition stages, in particular, within the ventromedial temporal allocortices and pro-isocortices and later temporoparietal neocortices (Tables 3, 4 and Figure 4). The effects of both pathologies differ to a larger extend, for instance, in the specificity to AD, to neurodegeneration in general, or to cognitive functions (Van Hoesen and Solodkin, 1994). However, the “macro sequence” of archicortex—mesiotemporal cortex—temporoparietal neocortex is the same, and the impaired cognitive domains in AD (memory and visuoconstruction) are associated with those regions.

### Neurotransmitters

Of interest in AD pathogenesis are especially two transmitter systems: the cholinergic and the glutamatergic systems. Acetylcholine is one of the essential neurotransmitters in the brain. Its functions are pleiotropic: acetylcholine is a fundamental transmitter in the peripheral vegetative nervous system and neuromuscular transmission. In the brain, acetylcholine is involved in many functional systems but particularly involved in the modulation of synaptic signaling (Van der Zee et al., 2011). The dysfunction of the cholinergic system is relevant in the pathogenesis of AD, as acetylcholine is essential for memory consolidation (Ferreira-Vieira et al., 2016). Anti-dementia drugs work as inhibitors of the acetylcholine esterase, increasing the concentration of acetylcholine in the synaptic gap, leading to slightly improved memory function (Ferreira-Vieira et al., 2016). Cholinergic effects have been shown to be involved in learning processes in the hippocampal formation by the enhancement of synaptic modification and selective presynaptic inhibition of synaptic transmission in different regions and layers (Hasselmo and Schnell, 1994). The beneficial effect of acetylcholine on memory encoding is probably mediated by strengthened synaptic modification, afferent input and spiking behavior (Hasselmo, 2006). On a functional level, cholinergic modulation has been linked to working memory for novel stimuli (Hasselmo and Stern, 2006). It has been hypothesized that synaptic connections exist for previously familiar stimuli (such as words or numbers) (Hasselmo and Stern, 2006), which makes working memory of these stimuli independent of cholinergic modulation (Crow and Grove-White, 1973; Broks et al., 1988). Moreover, acetylcholine is involved in excitability modulation in AD. While memory performance during task functional MRI can be correlated with activation of medial temporal lobe regions as the hippocampus and gyrus parahippocampalis, it was shown that stronger recruitment of those regions is associated with cognitive decline in MCI patients (Dickerson et al., 2004). The underlying hypothesis stated that hyperactivation could be seen as a compensatory effect due to hippocampal atrophy (Dickerson et al., 2004). Similarly, task functional MRI in cognitively still unimpaired *PSEN1* mutation carriers revealed increased activation of the right anterior hippocampus compared to non-carrier controls, many years before the estimated disease onset of familial AD (Quiroz et al., 2010). This can be brought into context with the role of cholinergic suppression in learning and AD. Runaway synaptic modification describes the phenomenon of exponential gain in synaptic connection strength, caused by activity evolving across already strengthened connections (Hasselmo, 1994). It can be seen as a natural consequence of Hebbian rules (Morris, 1999), but it interferes with learning processes, wherein only a selective subset of connections should be strengthened (the pattern to learn), while other existing strong connections should remain stable (Hasselmo, 1994). Cholinergic presynaptic inhibition of transmission along associative fibers offers a mechanism to protect from runaway synaptic modification during learning (Hasselmo and Bower, 1992). However, when hyperactivity in AD is introduced, it leads to more runaway synaptic modification, while vice versa, the strengthening of undesired networks can lead to more hyperactivity in a vicious circle (Hasselmo, 1994). The continuous presence of hyperactivation can further lead to excitotoxic effects (Hynd et al., 2004). Excitotoxicity refers to calcium-mediated toxic effects due to a sudden increase in glutamatergic transmission. Ashford and Jarvik hypothesized already in 1985 a preferential affection of highly neuroplastic connections with neurofibrillary tangles (Ashford and Jarvik, 1985), which has been further supported by a wide range of genetic and environmental AD risk factors that are associated with increased plasticity (Mesulam, 2000).

The concept of excitotoxicity is moreover essential for other transmitter systems as glutamate. Anti-dementia drugs that are no inhibitors of the acetylcholine esterase—mainly the N-methyl-D-aspartate (NMDA) receptor antagonist memantine—decrease glutamatergic transmission in the synaptic cleft. Glutamatergic dysfunction is also related to neuroinflammation and plasticity.

### Neuroimmunology

Besides the cascades of Abeta and Tau, another important hallmark in AD pathogenesis is the role of neuroinflammation and autoimmunity. One of the difficulties in understanding AD pathogenesis is that intrinsic proteinopathic dysfunction alone does not necessarily lead to neurodegeneration and cognitive decline. Such impairments are more likely to be caused by various toxic intermediate mechanisms, as discussed before. One potentially important but poorly understood mechanism is neuroinflammation. Neuroinflammation is a relevant factor in the pathogenesis of dementia because it is always the last part of the pathogenic cascade and leads directly to neuronal death (Heneka et al., 2015a). However, it is not clear how the modulation of inflammation can affect the process of neurodegeneration. Clinical trials have shown contradictory results. For instance, the long-term administration of non-steroidal anti-inflammatory drugs showed positive preventive effects and can hence reduce the *a priori* risk for AD (Wang et al., 2015). However, in contrast to those observational studies, prospective trials with steroids and other immunosuppressive drugs have not shown significant effects, and neither have randomized controlled studies with non-steroidal anti-inflammatory drugs (Jaturapatporn et al., 2012). A case-control study in patients with rheumatoid arthritis (who have a slightly higher risk for AD) showed a significant reduction of AD incidence by 70% (adjusted Odds ratio of 0.30, *p* = 0.02) if the patients were treated with the tumor necrosis factor α inhibitor etanercept (Chou et al., 2016), as long as it was well tolerable (Butchart et al., 2015). As the etiology and differential diagnosis of dementia is often unclear, the label of AD could cover up a relevant percentage of autoimmunological neural phenomena that could be treated with high-dose and long-time corticosteroid therapies (Pruss and Lennox, 2016). Cerebral immunology is complex, as it involves the organ-specific immunological cell type of microglia. An appropriate discussion would go beyond this review's scope, and we would like to refer the interested reader to the following review on neuroimmunology and AD (Heneka et al., 2015c).

### Imaging

#### Anatomical Magnetic Resonance Imaging

MRI offers a commonly used technique to screen for biomarkers *in vivo*. As described, the pathogenetic pattern of AD consists of the accumulation of amyloid plaques and neurofibrillary tangles. Volumetric assessment of gray matter loss in MRI has been identified to correlate with the distribution and degree of neurofibrillary tangle accumulation (Csernansky et al., 2004; Whitwell et al., 2008). Therefore, volumetric MRI can provide a proxy measurement for regional neurofibrillary tangle load (Persson et al., 2017).

AD patients have consistently been found to have atrophy of memory-related structures, including the hippocampus and other mesiotemporal regions, as well as the precuneus, cingulate, and the prefrontal areas (Braak and Braak, 1991; Frisoni et al., 2002; Karas et al., 2004; Shiino et al., 2006; Rosenbloom et al., 2011). However, non-amnestic symptoms like aphasia, visuospatial problems, or behavior-predominant dysfunction are initially present in up to 30% of AD patients (Koedam et al., 2010; Dickerson et al., 2017). The distribution of neurofibrillary tangles in those patients with an atypical clinical presentation is either limbic-predominant, hippocampal-sparing, or not reported, which is also referred to as the no-atrophy or minimal-atrophy AD variant (Murray et al., 2011; Persson et al., 2017). A correlation between these phenomenological subtypes of AD and volumetric MRI has already been demonstrated (Whitwell et al., 2012). Multiple studies explored MRI as an *in vivo* marker of these AD subtypes (Byun et al., 2015; Hwang et al., 2016; Ferreira et al., 2017; Persson et al., 2017). Besides the atrophy patterns of syndrome variants in AD morphology, a few single-region-based volume reductions have also been identified as potential biomarkers for AD. A detailed overview of these features is provided in Table 5.

**Table 5:** Overview of brain imaging studies and their results in Alzheimer's disease for different modalities.

**Changes in AD imaging compared to healthy controls**		**Imaging modality**	**Reference for evidence**	**Contradicting evidence**
Global connectome changes	Decreased global efficiency/longer characteristic path length	EEG	(Stam et al., 2007; de Haan et al., 2009)	
		MEG	(Stam et al., 2009)	
		fMRI	Amnestic MCI (Minati et al., 2014); AD (Sanz-Arigita et al., 2010; Zhao et al., 2012)	Similar characteristic path length as controls (Supekar et al., 2008)
		sMRI	(Lo et al., 2010; Reijmer et al., 2013; Daianu et al., 2015; Zhao et al., 2017) (not significant); (He et al., 2008; Yao et al., 2010)	
	Decreased averaged local efficiency	sMRI	(Reijmer et al., 2013)	Increased averaged local efficiency in fMRI (Zhao et al., 2012)
	Decreased global clustering	EEG	(de Haan et al., 2009)	
		MEG	(Stam et al., 2009)	Preserved clustering coefficient in EEG (Stam et al., 2007)
		fMRI	Amnestic MCI (Minati et al., 2014); AD (Supekar et al., 2008; Dai et al., 2019)	Increased global clustering in fMRI (Zhao et al., 2012); unchanged global clustering in fMRI (Sanz-Arigita et al., 2010)
		sMRI	(Reijmer et al., 2013; Pereira et al., 2016; Dai et al., 2019) (not significant)	Increased clustering coefficient in structural MRI (He et al., 2008; Yao et al., 2010);
	Decreased network robustness	MEG	(de Haan et al., 2012)	
	Altered modular structure	fMRI	Amnestic MCI (Minati et al., 2014); AD (Chen et al., 2013; Dai et al., 2019)	
		sMRI	(Pereira et al., 2016; Dai et al., 2019)	
	Rich club organization affected	sMRI	(Pereira et al., 2016; Yan et al., 2018; Dai et al., 2019)	
Network changes	DMN is attacked by AD	fMRI	(Çiftçi, 2011; Hahn et al., 2013; Dai et al., 2014, 2019; Bernard et al., 2015; Chen et al., 2016; Cope et al., 2018)	Increased local efficiency in the DMN in fMRI (Zhao et al., 2012)
		sMRI	(Hahn et al., 2013; Zhao et al., 2017; Dai et al., 2019)	
	The core of the network is most affected	sMRI, MEG, and fMRI	(Guillon et al., 2019)	Predominantly low-degree regions outside the core loose connectivity in structural MRI (Daianu et al., 2015)
	Increased connectivity for sensorimotor system	sMRI, MEG, and fMRI	(Guillon et al., 2019)	
Regional connectome changes	Decreased connectivity in the insula	fMRI	(Chen et al., 2013)	
	Decreased connectivity in the posteromedial cortex	fMRI	(Xia et al., 2014)	
	Decreased connectivity in the medial temporal cortex	fMRI	(Burggren and Brown, 2014)	
	Decreased connectivity in the amygdala	fMRI	(Yao et al., 2013; Wang et al., 2016)	
	Decreased connectivity in the parahippocampal area	sMRI	(Solodkin et al., 2013)	
	Decreased connectivity in frontal regions	sMRI	(Lo et al., 2010)	Increased connectivity within frontal areas in fMRI (Supekar et al., 2008)
	Disconnection of the precuneus, parietal and temporal areas	fMRI	Amnestic MC (Minati et al., 2014)	
	Reduced local clustering for the hippocampus	fMRI	(Supekar et al., 2008)	
	Decreased connectivity within the temporal lobe	fMRI	(Supekar et al., 2008)	
Regional atrophy	Atrophy in the hippocampus	sMRI	Mild dementia stage of AD (Bosscher and Scheltens, 2002; van der Flier et al., 2005); amnestic MCI (Shi et al., 2009)	
	Atrophy and thinning of the entorhinal cortex	sMRI	(Bobinski et al., 1999; Dickerson et al., 2001; Teipel et al., 2006; Velayudhan et al., 2013; Blanc et al., 2015)	
	Reduction of amygdala volume	sMRI	(Whitwell et al., 2005; Barnes et al., 2006)	
	Volume loss in the thalamus	sMRI	(Callen et al., 2001; Yi et al., 2016)	
Tau PET	Reduction in caudate nucleus volume	sMRI	(Rombouts et al., 2000; Madsen et al., 2010)	
	Atrophy in the nucleus accumbens	sMRI	(Liu et al., 2010; Yi et al., 2016)	
	Global neocortical Tau binding increased	^18^F-AV-1451	(Cho et al., 2016; Pontecorvo et al., 2019)	
	Early Braak stage Tau binding increased	^18^F-AV-1451, ^11^C-PBB3	Entorhinal cortex in MCI (Cho et al., 2016); Precuneus and lateral parietal in inherited AD (Gordon et al., 2019); lateral and medial frontal cortex in AD (Harrison et al., 2019); orbitofrontal cortex in AD (^11^C-PBB3) (Kitamura et al., 2018); middle to high Braak stages (Schöll et al., 2016)	Increased Tau binding in older healthy controls' temporal and retrosplenial cortex (Harrison et al., 2019)
	Tau in network hubs	^18^F-AV-1451, ^11^C-PBB3	(Cope et al., 2018; Kitamura et al., 2018)	Low consistency between atrophy and Tau deposition in atypical AD (^18^F-AV-1451) (Sintini et al., 2019)
Abeta PET	Global Abeta binding increased	^18^F-AV-45 (Florbetapir)	Visual rating (Clark et al., 2011; de Wilde et al., 2018)	
	Early Braak stage Abeta binding increased.	Florbetaben (^18^F), ^11^C-PIB	Inferior frontal cortex and precuneus (Alongi et al., 2019); striatum in hereditary *PSEN1* patients (Klunk et al., 2007) and *PSEN1*/*APP* patients (Villemagne et al., 2009); (Murray et al., 2015)	Middle to high Braak stages in HC, but age-related and associated with ApoE: with ^11^C-PIB (Jack et al., 2014), meta-analysis (Jansen et al., 2015); medial temporal lobe in HC (Song et al., 2015)
	Abeta binding increased in DMN	^18^F-AV-45 (Florbetapir)	Hubs of DMN including hippocampus (Chang et al., 2015)	
Glucose PET	(left) temporoparietal hypometabolism	^18^FDG	Left precuneus, posterior cingulate and superior parietal cortex in MCI-to-AD converters (Morbelli et al., 2010), bilaterally in Fukai et al. (2018), and ApoE ε4 carriers (Langbaum et al., 2010); temporal, angular and posterior cingular areas (Ou et al., 2019); frontal, posterior temporal, and parietal cortex (Meltzer et al., 1996)	Age-related temporal hypometabolism in HC (Jack et al., 2014); low sensitivity in a meta-analysis for differentiation of MCI converters (Smailagic et al., 2015)
	Hypometabolism associated with Tau	^18^FDG	Hypometabolism only in the presence of Abeta in Tau-positive regions (Adams et al., 2018)	Hypermetabolism caused by low Tau burden in the absence of Abeta (Adams et al., 2018)

*EEG, electroencephalography; MEG, magnetoencephalography; fMRI/sMRI, functional/structural magnetic resonance imaging; PET, positron emission tomography; DMN, default mode network; AD, Alzheimer's Disease; MCI, mild cognitive impairment*.

As a non-invasive *in vivo* measurement, MRI opens up the possibility of longitudinal tracking of atrophy and disease progression of AD. Recent studies investigated volume loss in AD patients longitudinally (Harrison et al., 2019; Pontecorvo et al., 2019; Sintini et al., 2019). Regions with reduced baseline gray matter volume also tend to show more atrophy over time and the highest atrophy rates are in the temporoparietal regions (Sintini et al., 2019).

Besides gray matter atrophy, white matter hyperintensities, which appear on T2-weighted or fluid-attenuated inversion recovery MRI scans, have a high prevalence among AD patients (Brickman, 2013). White matter hyperintensities, in general, can be morphological correlates of microvascular lesions as well as inflammatory or unspecific changes in aging. Increased overall hyperintensity volume has been observed 6–22 years before estimated symptom onset of AD (Lee et al., 2016). The relationship between white matter hyperintensities and AD pathology is still an active research field (Graff-Radford et al., 2019).

#### Positron Emission Tomography

Nuclear imaging methods allow *in vivo* acquisition of metabolic features of the brain by using various radioactively marked tracer molecules, so-called radionuclides. PET offers a remarkable possibility of different functional assessments of the brain. The underlying procedure makes use of β^+^-emitters: the emitted positrons of β-decay react with electrons of the tissue in a so-called annihilation. This leads to the emission of photons, which can be measured by specific sensors (Phelps, 2000).

Both Abeta and Tau deposits can be detected indirectly by PET and correspond well to the underlying pathologic changes at autopsy (Clark et al., 2011; Schöll et al., 2016). Tau tracer binding in AD is not only increased in regions that are known to be affected in early Braak stages (Cho et al., 2016; Schöll et al., 2016; Kitamura et al., 2018; Gordon et al., 2019; Harrison et al., 2019), but also globally heightened in the cortex (Cho et al., 2016; Pontecorvo et al., 2019). However, Tau binding is also present in healthy controls, predominantly located in areas with atrophic changes (Harrison et al., 2019). Similarly, Abeta tracers show increased global deposition in the whole brain (Clark et al., 2011; de Wilde et al., 2018) and in early Braak stage regions (Murray et al., 2015) (Alongi et al., 2019). However, the percentage of “Abeta-positive” healthy controls seems higher than for Tau (Jack et al., 2014; Jansen et al., 2015; Song et al., 2015).

Another important PET measurement is the assessment of energy metabolism by the usage of marked glucose molecules. Multiple studies suggest temporoparietal hypometabolism in AD (Meltzer et al., 1996; Langbaum et al., 2010; Morbelli et al., 2010; Fukai et al., 2018; Ou et al., 2019), which is already an established marker for unclear cases of other dementias in clinical practice. Interestingly, similar to atrophy patterns (Csernansky et al., 2004; Whitwell et al., 2008), hypometabolism has a strong association with Tau deposits (Adams et al., 2018). The usage of glucose PET in clinical routine is limited by its high costs, exposure to ionizing radiation, and low sensitivity in detecting MCI patients that will convert to AD (Smailagic et al., 2015).

An overview of PET findings in AD is provided in Table 5.

#### Connectomics

After discussing the recent advances researching the microscopic molecular level in AD, we consider a whole-brain perspective at the macroscopic brain region level. The connectomic approach is a neuroscientific discipline that analyzes, describes, and uses (axonal) connectivity measures of the brain (Fornito et al., 2015). It provides an overview of the disease effects in AD and identifies global phenomena beyond the impairment of single regions.

**Figure 6:**
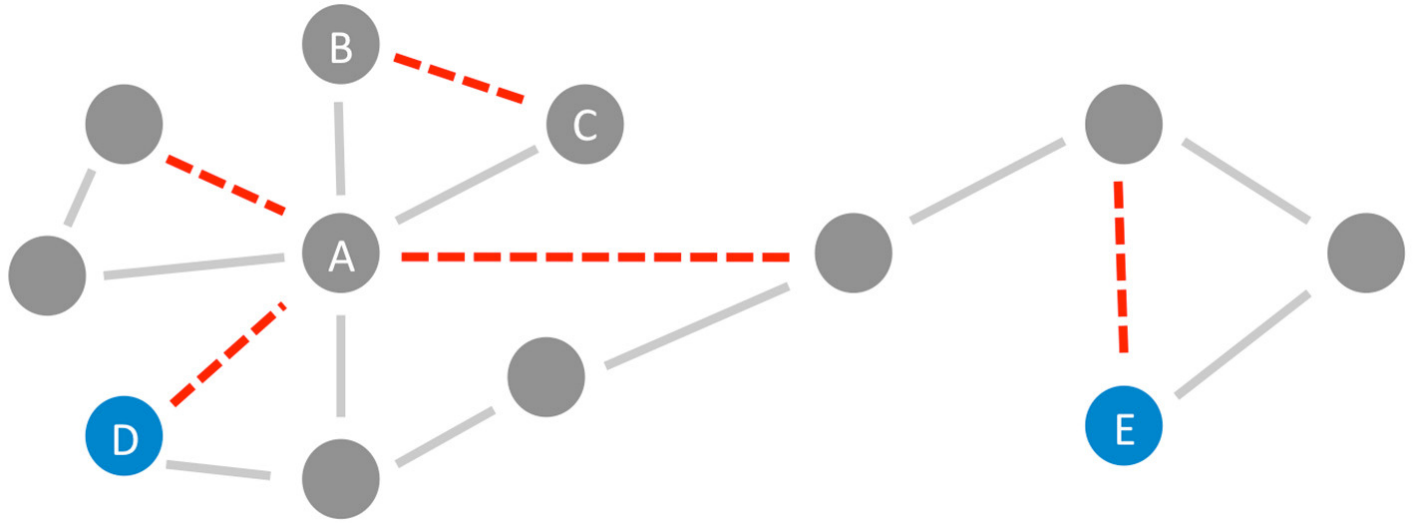
Neurodegeneration in Alzheimer's Disease (AD) from a network perspective. In this schematic example network, the red links (edges) are being weakened and progressively disconnected by AD. Preferentially, edges attached to nodes with high degree (hubs) are being targeted (here node A) (Stam et al., 2009; Lo et al., 2010; Yan et al., 2018). Besides, lower clustering in AD has repeatedly been observed (Brier et al., 2014b; Minati et al., 2014; Pereira et al., 2016; Dai et al., 2019), i.e., links involved in triangles are broken off (here, e.g., the link between nodes B and C forming the triangle A-B-C). These two “attacks” of AD on the network lead not only to a lower clustering coefficient but also evoke a lower efficiency, defined here as the inverse of the global path length. This lower efficiency is demonstrated in the example network by the shortest path length between the blue nodes D and E before and after the deletion of the red links (before: 4 links, after: 6 links).

In the general framework of brain networks, regions are represented by nodes, and connections between them (either structural or functional) are denoted as links or edges (Figure 6 shows an abstract example network). At this level of abstraction, it is possible to calculate graph-theoretic measures for the connectome, so-called network metrics (Bullmore and Sporns, 2009). A plethora of different—partly interdependent—metrics shows changes in AD networks compared to healthy controls. An overview of these findings is provided in Table 5. Heterogenous findings for different measurement modalities exist, pointing toward a widely spanned network disruption in AD on different scales (Dennis and Thompson, 2014; Stam, 2014). This heterogeneity can also be explained by different methodological choices for network construction, e.g., setting different thresholds for filtering out the most essential connections (van Wijk et al., 2010; Tijms et al., 2013; van den Heuvel et al., 2017). In general, however, converging evidence suggests aberrant functional connectivity (measured by functional MRI) and abnormal white matter structural connectivity in AD compared to healthy aging. We review and align the recent literature on this topic showing multiple local network changes resulting in the global phenomena of less efficient network communication for AD patients compared to healthy controls.

Connectomic research provides an important perspective for understanding the development of cognition and its decline in dementia. There are various hypotheses about the network changes in dementia highlighting different aspects of neurodegeneration (Dennis and Thompson, 2014). One line of research refers to dementia as a disconnection syndrome (Brier et al., 2014a), where the loss of neurons and small-scale connectivity influences the macro-scale in the form of (structurally and functionally) disconnected brain areas (Delbeuck et al., 2003; Stam, 2014). This disconnection correlated with the cognitive and behavioral decline (Stam, 2014) and white matter pathology in certain areas could be used as a biomarker for disease progression (Solodkin et al., 2013). This view on AD as a disconnection syndrome was able to bridge multiple scales of disease pathology in a coherent way. In recent years, however, network science studies on AD patients expanded this picture: widespread increases and decreases of connectivity within the brain network were observed, pointing toward compensatory mechanisms or reactions of the network beyond disconnection (Stam, 2014). Even an early but seminal study conveying the importance of “small networks studies,” especially related to the initial disease stages, showed isolation of the hippocampus from its cortical network connections through initial entorhinal cortex degeneration *via* the perforant pathway (Hyman et al., 1984).

In network science, brain regions with high connectivity to other regions are called *hubs*. For both structural and functional connectivity studies, hub regions have consistently been identified as the most affected areas by AD (Stam et al., 2009; Lo et al., 2010; Yan et al., 2018). De Brier et al. showed that even in preclinical stages of AD, hubs are disrupted, where hubs were defined here as nodes with the highest betweenness centrality (a measure for involvement in important pathways) and the highest participation coefficient (in how far the node is connected to different modules or subnetworks) (Brier et al., 2014b). This vulnerability of hubs correlates with a higher Abeta burden in these hub regions (Cope et al., 2018). A focus of the disconnection in AD lies on the default mode network, a large-scale network of regions strongly interconnected in resting state (Çiftçi, 2011; Hahn et al., 2013; Dai et al., 2014, 2019; Bernard et al., 2015; Chen et al., 2016; Cope et al., 2018). Although this phenomenon is also observed in aging (Perry et al., 2015), the degree of default mode network disruption allows in parts the distinction between healthy aging and AD (Greicius et al., 2004). Regarding the functional network, especially the default mode network is targeted by the AD-caused neurodegeneration (Çiftçi, 2011; Hahn et al., 2013; Dai et al., 2014, 2019; Bernard et al., 2015; Chen et al., 2016), where the highest Abeta deposition is also located. The extent of hub disruption correlates significantly with the cognitive status of a patient (Dai et al., 2014). Thus, it can be hypothesized that hubs—with their high Abeta deposition and central role in the overall information flow of the brain network—facilitate the spreading of the pathological cascade within the brains of AD patients (Buckner et al., 2009). Aberrant or decreased functional connectivity has also been observed in the insula (Chen et al., 2013), posteromedial cortex (Xia et al., 2014), medial temporal cortex (Burggren and Brown, 2014), and amygdala (Yao et al., 2013; Wang et al., 2016).

Next to the vulnerability of hubs, a decreased global clustering coefficient has been reported, showing a loss of connectedness and important redundancy structures for brain communication in FC, which consequently also alters the modular structure of AD patients (Brier et al., 2014b; Minati et al., 2014; Pereira et al., 2016; Dai et al., 2019). Probably as a global effect of these “local attacks” on the network (Figure 6), decreased global efficiency in structural connectivity as well as functional connectivity networks is often observed in AD patients, which correlates with cognitive and behavioral decline (Lo et al., 2010; Reijmer et al., 2013; Dai et al., 2019). Global efficiency is in network science defined as the inverse of the characteristic path length—with shorter pathways between the nodes, the information flow within the network is more efficient. A less efficient network can still provide connections between nodes, but they are longer and with more nodes and edges in-between (Bullmore and Sporns, 2009).

Recent work analyzed a multimodal perspective on AD, combining diffusion tensor imaging, functional MRI, and magnetoencephalography measurements in a multilayer network (Guillon et al., 2019). They found that the core of this multilayer network, which is likely to contain the hubs, has been most affected, which establishes hubs' vulnerability across modalities. Together, these changes were able to predict the cognitive and memory impairment of patients (Guillon et al., 2019).

Emerging evidence suggests that in the preclinical stage of AD, network changes are present in the form of disconnection on a large scale (Brier et al., 2014b; Daianu et al., 2015; Zhao et al., 2017). Indeed, functional connectivity and structural connectivity are now being investigated to better differentiate AD patients, MCI, and controls, moving toward the goal of identifying prodromal AD patients and the possibility of developing early intervention strategies (Phillips et al., 2015; Pereira et al., 2016; de Vos et al., 2018; Ye et al., 2019). A recent study suggests altered functional connectivity corresponding to accelerated aging in preclinical AD (Gonneaud et al., 2020).

To sum up, both altered global and local connectivity have been associated with AD. Converging evidence from white matter diffusion tensor imaging and resting-state functional MRI studies point toward less efficient network communication in AD patients compared to healthy aging, especially in the default mode network.

## Modeling of Alzheimer's Disease

As AD is a complex disease that takes place on various scales, a wide range of models have been developed for its analysis, e.g., animal disease models (Saito et al., 2014; Weintraub et al., 2014), cognitive models (Sevush et al., 2003), or disease progression and classification models (Bhagwat et al., 2018; Khanna et al., 2018; Koval et al., 2018; Pellegrini et al., 2018; Golriz Khatami et al., 2019). Mathematical modeling is an adaptive and creative scientific concept and a core technique of computational neuroscience. In general, one can differentiate between approaches focusing on single aspects of the disease, e.g., biochemical Abeta modeling (George and Howlett, 1999), and integrative models incorporating several biomarkers while using multiple scales simultaneously (Khanna et al., 2018). The latter might provide a more comprehensive, multimodal view on the disease with its interacting mechanisms and might be more suitable to reflect disease pathogenesis. This multiscale approach—also called “integrative disease modeling” (Younesi and Hofmann-Apitius, 2013)—can combine functional and structural neuroimaging techniques, cerebrospinal fluid sampling, and genomic data and analyzes their intercorrelations with computational algorithms (Golriz Khatami et al., 2019).

A comprehensive understanding of both the underlying biological processes of AD and the computational framework of high-performance modeling approaches is necessary to develop novel models for AD that integrate multiple scales, modalities, and research disciplines. With increasing technical possibilities for high-performance computing and growing hierarchically organized knowledge architectures, this cross-disciplinary approach holds the potential to overcome some of the enigmas in AD pathogenesis that might not be revealed on a single scale applying a single method.

Therefore, in the following sections, we describe existing computational (brain) models of different scales and outline how far they can be linked to the biological concepts presented before.

### Statistical Disease Prediction Models

Statistical prediction models are mainly descriptive when used in linear classification tasks. Subjects are assigned to a diagnostic category (HC, MCI, or AD) based on the input data. But beyond their practical translational usage as diagnostic tools, prediction models provide certain decision criteria that can also be of interest in understanding the underlying disease mechanisms (Jack and Holtzman, 2013). In addition to those rather simplistic linear models there are methods such as machine learning (Moradi et al., 2015; Pellegrini et al., 2018) or Bayesian modeling (Khanna et al., 2018). All exhibit individual challenges and advantages regarding data analysis and model interpretation (Poil et al., 2013).

Machine learning approaches are applied to predict disease trajectories (predictive modeling) or classify subjects into groups with highly similar data points (discriminative modeling or clustering). The latter goal can be reached by supervised *a priori* labeling of the training data (e.g., as two classes AD and non-AD, or as a three-class problem with AD, MCI, and healthy controls) or by unsupervised clustering without labeling (Golriz Khatami et al., 2019). Those unsupervised discriminative models cluster subjects based on the degree of (dis-)similarity between parameters. This can be quantitatively expressed by statistical proximity measures (Bock, 2005; Golriz Khatami et al., 2019).

Structural T1-weighted MRI, in conjunction with other biomarkers, has been considered as a feature for classic machine learning techniques, such as support vector machines—often combined with linear discrimination analysis. Pellegrini et al. (2018) reported in a review that, while patients with AD could successfully be differentiated from controls, the classification of subjects with MCI remained unsatisfactory (Pellegrini et al., 2018). This held also true for the risk prediction of conversion from MCI to AD. Thus, the classifiers' clinical relevance remains relatively low, given that—in practice—it is already possible to distinguish between controls and AD based on cognitive performance. A diagnosis before clinically-noticed AD onset is therefore still missing.

Attempts of facilitating early AD prediction are therefore building biologically informed models that develop “mechanistic biomarkers” by aiming at a deeper understanding of AD pathomechanisms (Selkoe, 2004). To achieve that, a complex disease knowledge system (i.e., ontology) can be built from different data sources, and biologically plausible predictors are deduced. This contrasts the approaches described above in which the selection of biomarkers is mainly based on statistical dependence (e.g., correlations). Mechanistic biomarkers however resemble biologically plausible concepts instead of merely relating to the disease by correlation.

Using this approach, biological mechanisms of the transition from asymptomatic stages or MCI to AD have been computationally reconstructed to achieve a more accurate risk prediction (Khanna et al., 2018). By using a predictive time-to-event model that incorporates multimodal data ranging from genetic variants to neuroimaging and neuropsychological assessments, several biological risk factors and their interaction could be extracted (Khanna et al., 2018). As the model makes use of a graph-like Bayesian network organization (Khanna et al., 2018), it opens up new possibilities for the integration of multiscale and multimodal information to discover more possible mechanistic biomarkers.

Additionally, information about disease progression can be extracted from longitudinal patient data to increase the accuracy of subsequent predictions. For example, Bhagwat et al. (2018) have modeled AD disease trajectories of patients with varying cognitive performance at baseline by combining longitudinal data of MRI brain volumetry (cortical thickness) and clinical assessments with genetic information (ApoE ε4 status). Comparing different algorithms trained on multimodal data from two time points, a longitudinal predictive neural-network showed the highest performance, even after validation with a second untrained data set (Bhagwat et al., 2018).

This longitudinal and multimodal approach of predicting the individual risk and disease trajectories could thus represent promising new paths in personalized medicine. Modeling structural and metabolic changes in different brain areas concerning the decline in cognitive functions can yield more sophisticated information about disease progression and its influencing factors on an individual level (Koval et al., 2018). Future approaches could also incorporate a broader range of data modalities from different sources, like the graph-theoretically-organized database European Brain Research Infrastructures (EBRAINS) of the Human Brain Project (https://ebrains.eu, Markram et al., 2011). EBRAINS hosts detailed data for many brain areas from a variety of modalities, such as receptor densities (Palomero-Gallagher and Zilles, 2018) or gene expressions (Yetman et al., 2016). Besides that, the Multimodal Mechanistic Signatures Database for Neurodegenerative Diseases (NeuroMMSig, https://neurommsig.scai.fraunhofer.de, Domingo-Fernández et al., 2017) poses great potential for mechanistic models. NeuroMMSig, a hierarchically organized ontology, integrates chemical compounds, genes, proteins, medical terms, and imaging features into a mechanistic pathway representation of AD. These pathways (i.e., cause-and-effect chains of biological concepts or processes) were retrieved by methods of literature mining and condensed into 125 sub-networks that play a distinct role in the pathophysiology of AD (Domingo-Fernández et al., 2017). By integrating big databases like those described, promising new approaches for the predictions of individual disease trajectories with mechanistic cause-and-effect models are posed.

### Sub-cellular Models

Brain simulation can occur on many different scales, as the complex topological hierarchy of the brain consists of many essential components: cortical and subcortical regions, networks, columns, ensembles, circuits, neurons, synapses, vesicles, molecules, and genes (compare Figure 5).

Sub-cellular features of AD provide promising input for computational modeling based on protein interaction and gene expression. Early AD modeling approaches have focused e.g., on the deposition process of Abeta (Jarrett et al., 1993; Lomakin et al., 1997; Pallitto and Murphy, 2001; Ortega et al., 2013). Moreover, biochemical models account for the interaction between numerous factors like Abeta, Tau, inflammation, and different proteases, as well as possible interventions during the disease course (Proctor and Gray, 2010; Anastasio, 2013, 2014; Kyrtsos and Baras, 2013; Proctor et al., 2013).

Early studies have used computational modeling to assess aggregation kinetics for synthetic Abeta-like peptides (Tomski and Murphy, 1992). Comparably simple biochemical models allowed a mathematical description of the aggregation process—as the temporal evolution of Abeta in the form of monomers, micelles, and fibrils (Lomakin et al., 1997). Subsequently, the Abeta aggregation theory was enhanced by including more detailed interactions between different forms of Abeta fibrils and fitting the model to empirical data (Pallitto and Murphy, 2001). As experimental evidence on Abeta's toxicity increased, a particular model was developed that describes disrupted Ca^2+^ homeostasis and Abeta aggregation as a positive feedback loop and their interaction in a vicious circle (De Caluwé and Dupont, 2013). Over the last decade, more specific models have included associations of AD to important gene transcription factors as p53 (Proctor and Gray, 2010), possible intervention strategies (Proctor et al., 2013), and genetic risk factors (Kyrtsos and Baras, 2013).

Concepts of sub-cellular modeling are valuable for integrating multiscale models as they describe the molecular hallmarks of AD in a computationally accessible manner. Molecular pathways can be “coded” as a network of relations by employing computational linguistics and semantic frameworks. One possible tool for this approach is the Biological Expression Language (BEL), which makes it possible to describe the interaction between proteins, genes, and other chemical compounds with means of first-order logic (Madan et al., 2019).

### Single-Neuron and Neural-Circuit Models

Besides the subcellular scope, AD models span over different microscopic scales, ranging from single-cell models (Morse et al., 2010; Romani et al., 2013; Bianchi et al., 2014; Perez et al., 2016) to neural circuits (Zou et al., 2011; Abuhassan et al., 2012; Bianchi et al., 2014; Rowan et al., 2014).

Single-neuron models are often inspired by an experimental approach, such as a patch-clamp experiment (Chen, 2005), in the effort of reproducing the observed data (Morse et al., 2010). Underlying mathematics for those single-cell simulations may refer to general formulations for neural oscillation models, as it is the case for Hodgkin-Huxley model (Hodgkin and Huxley, 1952). Hodgkin and Huxley delivered the first impactful mathematical description of electric conductances in a neuron model in 1952 (Hodgkin and Huxley, 1952). The Hodgkin-Huxley model is based on experimental recordings on squid axons: By defining the phospholipid membrane's capacitance and the conductance of leak and voltage-gated ion channels, it enables a realistic approximation of membrane potentials over time (Hodgkin and Huxley, 1952). However, the model is computationally expensive, qualifying it mainly for simulations with either few neurons or small simulation length (Izhikevich, 2004). There seems to be a dilemma between biologically plausible but comparably inefficient models (e.g., Hodgkin and Huxley, 1952; Morris and Lecar, 1981; Rose and Hindmarsh, 1989; Wilson, 1999) and very efficient models that lack plausibility as they show a limited range of possible behaviors (e.g., the integrate-and-fire or integrate-and-fire-or-burst model; Smith et al., 2000; Izhikevich, 2004). A possible solution was supposed by Izhikevich, providing a computationally efficient model with the ability to produce emergent biological phenomena as tonic and phasic spiking and bursting, frequency adaptation, and accommodation (Izhikevich, 2003, 2004).

The mean-field theory can integrate complex systems of a large number of neurons (Spiegler et al., 2011). The simplification of the mean field has its origin in physics to describe fluid or gas behavior without considering individual molecules. In the brain, it allows simplifying the behavior of a spatially distinct group of neurons with a similar function (Liley et al., 2002). This group of neurons is called a neural mass and can be defined on various scales—e.g., as a brain region, a column, or a neuronal ensemble. Neural mass models (Wilson and Cowan, 1972; Zetterberg et al., 1978; Hindmarsh and Rose, 1984; Jansen and Rit, 1995; Wong and Wang, 2006; Stefanescu and Jirsa, 2008; Sanz-Leon et al., 2015) have been widely used to define local dynamics in a large-scale brain network model.

### Large-Scale Brain Network Models

The evolution of large-scale computational brain modeling has accelerated over the past decade. de Haan et al. (2012) built a model to test the hypothesis that excessive neural activity leads to neurodegeneration. This model is a large-scale brain network derived from diffusion MRI, where each network node holds a neural mass model by Zetterberg et al. (1978) as its local dynamic model. De Haan and colleagues simplified the synaptic strength as a function of neural activity over time (de Haan et al., 2012). As a result, those connections transmitting higher activity became weakened after a certain time period. The purpose of implementing this specific mechanism was to describe a form of excitotoxicity that leads to degeneration. Here, after a certain period, one could consecutively observe degeneration in the functional and structural network topology using graph-theoretical measures. The authors also observed a loss of spectral power and an increased sensitivity of hubs, defined as highly connected brain regions (incoming and outgoing ties) (de Haan et al., 2012). The authors observed an increase in brain activity and functional connectivity in the model, similar to empirical findings in MCI or mild AD stages (de Haan et al., 2012). A subsequent study by de Haan et al. (2017) tested different “therapeutic” strategies, like increasing or decreasing the excitability of excitatory and inhibitory subpopulations of the neural masses to prevent neurodegeneration in the excitotoxic model. The most convincing strategy, which could maintain healthy network features over a long time, was increasing excitability of excitatory neurons followed by increasing inhibition of inhibitory neurons. At first glance, this might seem contradictory, but it suggests the reversal of hyperexcitability by either more excitation or less inhibition. The authors suggested that the reason for this phenomenon might be in the network topology. The best strategies suppress the network hub activities, which in return may lead to decreased disease propagation. According to this prediction, neurodegeneration spreads along the network infrastructure as a kind of “pro-degenerative” signaling pattern. This can be related to an earlier description of Hasselmo in 1994. This model (Hasselmo, 1994) provides a descriptive model of runaway synaptic modification, learning, and cholinergic suppression that can explain essential findings of AD: the spatiotemporal pattern of disease progression along substantial fiber tracts, early memory deficits, and neurodegeneration due to excessive demands on synaptic plasticity rather than excitotoxicity. In contrast to the work by de Haan et al. (2012), which assumes neurodegeneration as a consequence of hyperactivation, the Hasselmo model (Hasselmo, 1994) explains an earlier part of the same process, wherein hyperactivation induces undesired neuroplasticity by extensive runaway synaptic modification and through this mechanism causes neurodegeneration and interferes with learning mechanisms.

Pons and colleagues used another brain network model for AD (Pons et al., 2010), using the neural mass model of Jansen and Rit (1995) at each cortical network node, which is related to the Zetterberg model (Zetterberg et al., 1978). The authors used electroencephalography recordings that showed a slowing of the alpha rhythm and an increase in functional connectivity (using phase lag index) in MCI patients with age, i.e., the functional connectivity increased from young to old subjects. Pons et al. were able to describe these observations by decreasing the maximum postsynaptic potential and increasing the thalamocortical SCs during simulations.

In another recent modeling study, Demirtaş and colleagues investigated the blood-oxygen-level-dependent (BOLD) signal changes due to AD (Demirtaş et al., 2017). This study included 109 subjects from different groups (healthy controls, preclinical AD, MCI, and AD). Regarding their empirical BOLD signal, one could observe a decrease in global interactions of AD patients evaluating first-order circular statistics, that is in the Kuramoto order parameter, as well as regional differences in the functional connectivity strengths, compared to the controls (Demirtaş et al., 2017). Further, functional connectivity differences were correlated to cerebrospinal fluid biomarkers like Abeta, total Tau, and phospho-Tau (Demirtaş et al., 2017). Estimating individual effective connectivity from subject-specific structural connectivity and functional connectivity with a heuristic approach, the brain model could replicate these observed changes (Demirtaş et al., 2017). A supercritical Andronov-Hopf bifurcation described its local dynamics. In an *in silico* experiment using brain network models based on healthy subjects' effective connectivity, Demirtaş et al. systematically varied the order parameter of the model (Demirtaş et al., 2017). In this way, they were able to observe the progress of functional connectivity degeneration. An optimal order parameter was individually found for each disease stage and group, replicating best the empirical observed degeneration. This study showed how changes in regional dynamics could lead to the disintegration of activity within the anatomical large-scale brain network. Concurrently, simulations also replicated the finding that the interaction (measured by Kuramoto order parameter) between BOLD signals declines with disease progression.

### The Virtual Brain Platform

In the following, we focus on The Virtual Brain, a multimodal and multiscale virtual brain simulation framework (Ritter et al., 2013; Sanz Leon et al., 2013; Sanz-Leon et al., 2015; Stefanovski et al., 2016; Solodkin et al., 2018) that holds the potential to combine different modeling scales of AD research. The open-source platform of The Virtual Brain is available under www.thevirtualbrain.org. The Virtual Brain is a standardized and an established framework that enables large-scale modeling approaches (as mentioned in the previous section Large-scale Brain Network Models) on individual patient data including a wide range of underlying dynamics.

The Virtual Brain uses the structural connectome as its underlying basis (Sanz-Leon et al., 2015). Most of the neural mass models (representing the regional activity) implemented in The Virtual Brain had their origin as a network model for smaller, distinct networks. But with the development of connectomics, the networks included were more complex and elaborate (Dipasquale and Cercignani, 2016). Likewise, the local dynamic models used in The Virtual Brain were, in principle, composed of smaller or even single-neuron systems (Wilson and Cowan, 1972; Zetterberg et al., 1978; Hindmarsh and Rose, 1984; Jansen and Rit, 1995; Wong and Wang, 2006; Stefanescu and Jirsa, 2008; Sanz-Leon et al., 2015). The Virtual Brain was designed to simulate whole-brain network dynamics, but it can also model and simulate separate subnetworks ranging from a regional level to a few neurons (see Spiegler and Jirsa, 2013 for the integration hierarchy of The Virtual Brain).

The second important feature of The Virtual Brain that can assist in AD research is the multiscale character. This term has been coined to describe the fluid transition of brain scales, ranging from the macroscale, at which brain regions interact intra- and inter-hemispherically *via* long-range connections, to the microscale of myriads of single neurons, where we have the knowledge on their electrophysiological properties, receptors, transmitters, position and wiring in cortical layers, etc. *In vivo* measurement techniques at the macroscale offer information about individual brains, whereas measurements at the micro-scale are more specific to cell membranes and structures but cannot sample an entire individual brain. The concept of The Virtual Brain addresses both scales: on the one hand, the structural connectivity of the whole brain is the scaffold of The Virtual Brain, and, on the other hand, the characteristics on the neural level are represented in the local dynamic models and their biophysiological parameters (e.g., the Jansen-Rit model). Modeling the large-scale brain alone may not comprise microscopical elements, as well as modeling the entire brain based on every single neuron may be computationally infeasible.

For this reason, the mesoscale has been established (Deco et al., 2008; Wright and Liley, 2010) and comprises different components. First, the direct electromagnetic fields between neighboring regions directly influence each other. In addition, neural masses, which can cover the anatomical extent of a functional region or cover the neural mass in a voxel sampled by an MRI scanner. Depending on the neural mass model, they can refer to excitatory and inhibitory populations interacting with each other and through the large-scale network, the connectome, with distant regions. The interplay of this local circuitry in the large-scale brain network can produce physiologically plausible brain activity on a large scale (Honey et al., 2007; Ghosh et al., 2008; Sotero and Trujillo-Barreto, 2008; Bojak et al., 2010; Jirsa et al., 2010; Ritter et al., 2013; Sanz-Leon et al., 2015; Kunze et al., 2016).

The prospect of The Virtual Brain as an interdisciplinary research framework is that clinical applications and ensuing technologies may benefit and build on theoretical and computational predictions, as it has already shown success in epileptic surgery (Jirsa et al., 2017; Proix et al., 2017). The Virtual Brain has already been used in a wide range of research topics, from the modeling of physiological brain phenomena in healthy participants (Ritter et al., 2013; Sanz Leon et al., 2013; Spiegler and Jirsa, 2013; Roy et al., 2014), mouse brain models (Melozzi et al., 2017), to clinical approaches of AD (Zimmermann et al., 2018; Stefanovski et al., 2019), stroke (Falcon et al., 2015, 2016), and brain tumors (Aerts et al., 2018).

A study by Zimmermann et al. modeled AD using The Virtual Brain (Zimmermann et al., 2018). By fitting the model to predict individual functional connectivity from the underlying structural connectivity, the authors could show a significant correlation between the cognitive state of AD patients and the fitted model parameters of The Virtual Brain (Zimmermann et al., 2018). As the parameters are surrogates of biophysically relevant entities such as long-range coupling factors and local interactions between inhibitory and excitatory neuronal populations, this enables the non-invasive estimation of intrinsic brain features.

For the field of AD, multimodal data could include, e.g., anatomical MRI, the structural connectivity out of diffusion tensor imaging, and PET imaging data of Glucose metabolism, Amyloid, and Tau. Our previous study (Stefanovski et al., 2019) used one of these features, namely Abeta PET, to explore the mechanisms behind another prominent phenomenon in AD: the slowing of electroencephalography (Stefanovski et al., 2019). As a pilot study in the field of molecular-driven large-scale brain simulations, we modeled local Abeta-mediated hyperexcitability using brain network modeling with The Virtual Brain, where regional Abeta burden was derived from PET data. By defining the local excitation-inhibition balance as a function of local Abeta burden from PET, fundamental differences between the AD patients and controls were observed. We showed that a few regions with moderate or high Abeta burden are transferred into an altered dynamic state, wherein their activity oscillations slowed down. This slowing is mainly presented as a shift from alpha to theta rhythm. It was propagated throughout the network and focused on the hubs. Interestingly, local hyperexcitation took also place in the central parts of the network. Therefore, with this approach, we were able to reveal a possible pathomechanism behind electroencephalographic slowing in AD (Stefanovski et al., 2019).

## Conclusions and Future Directions

**Figure 7:**
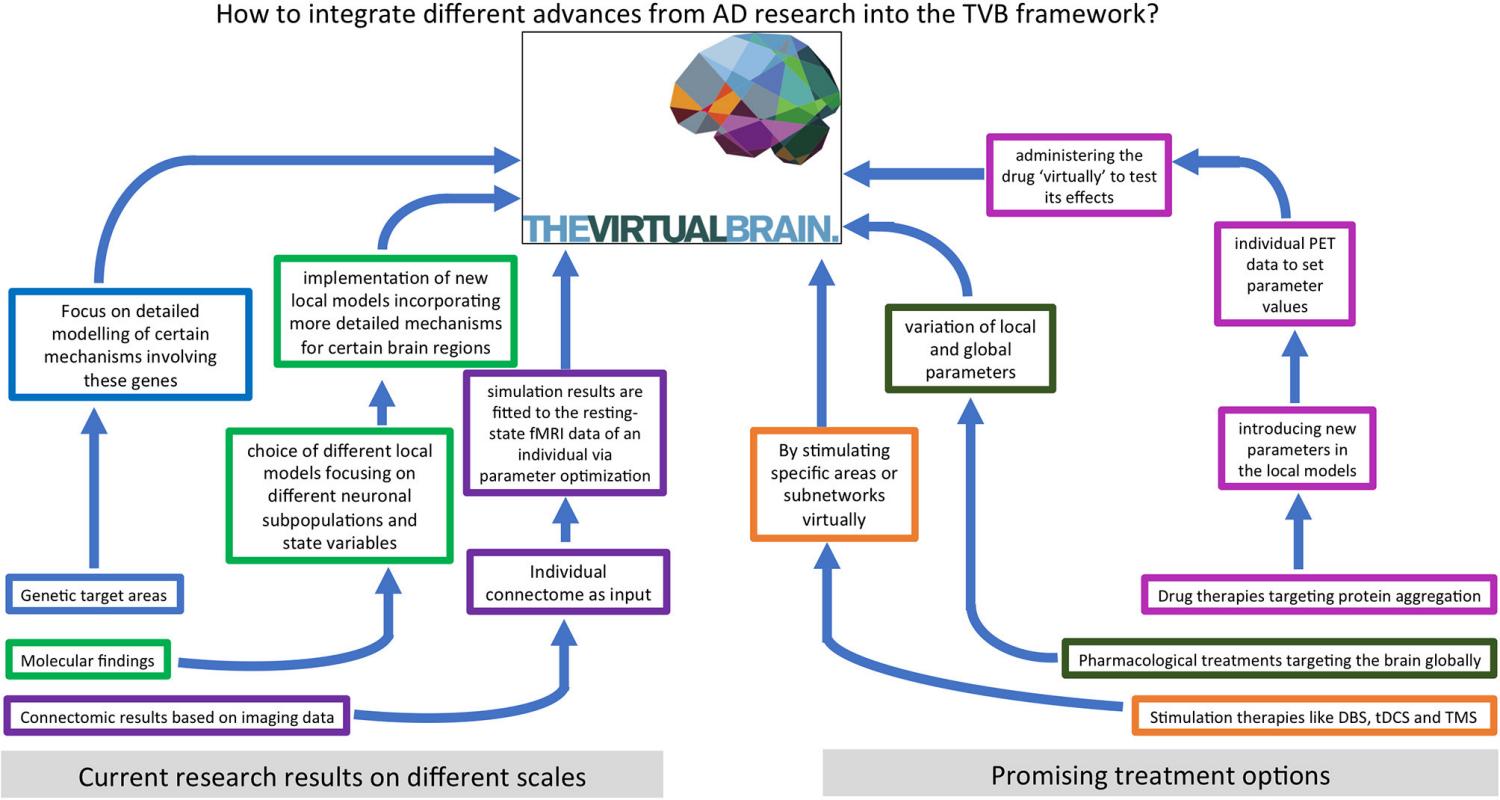
Potential applications of The Virtual Brain in the investigation of Alzheimer's Disease (AD). As we outline in this article, computational modeling provides a powerful tool to link empirical findings from different scales and disciplines to new insights for improved diagnostics and treatments. PET, positron emission tomography; DBS, deep brain stimulation; tDCS, transcranial direct current stimulation; TMS, transcranial magnetic stimulation.

Although our knowledge about the contributing factors in AD pathogenesis grows, it is still a major challenge in neuroscience to understand their distinct meaning and interaction. Moreover, the translation to clinical research is lagging behind. Rather than exploring isolated mechanisms, the goal should be to integrate multiscale datasets to reveal complex interactions underlying AD (Hofmann-Apitius et al., 2015; Iyappan et al., 2016) (Figure 7).

The main reasons for the development of precise and early diagnosis tools for AD can be summarized as follows:

1. Future treatments. When a disease-modifying treatment for degenerative dementia will be available, it probably needs to be performed many years before the clinical and behavioral manifestation of the disease. This is because pathway changes in the brain begin decades before the onset of dementia and lead to irreversible neuronal death. However, one has to expect that such a treatment has to be taken for many years and might have severe adverse reactions. Therefore, high sensitivity for the screening and high specificity of the diagnosis will be of crucial importance. Moreover, future trends of personalized treatments can only be performed with personalized biomarker-profiled patient 'fingerprints'. For example, a recent analysis has shown that the multimodal dataset from the Alzheimer's Disease Neuroimaging Database (ADNI) can predict the gene expression pattern (as a potential individualized treatment target) better than the clinical presentation does (Iturria-Medina et al., 2018).
2. Differential diagnosis. A more precise diagnosis of AD will lead to the possibility to clarify seemingly atypical cases. Some etiologies could be identified with prospective treatments. Moreover, in the future, subtypes of disease entities could be established, which are currently subsumed under AD, and assessed by a specific treatment, e.g., autoimmunity phenomena with immunosuppression or early-onset AD with anti-Abeta drugs.
3. Patient stratification. It is necessary to identify the right population to test new treatments. If the diagnosis is not clear enough, possible effects could be overlaid because of too many patients with other disease causes, in which the treatment does not show an effect.
4. Study monitoring. Because the disease's clinical trajectories are slow and not easy to measure objectively, it is beneficial to use biomarkers to monitor drug effects in study settings.

Using computational models for multiscale brain simulations in future research may lead to improved diagnostics in the early stages of dementia, to a more precise prognostic prediction and differential diagnosis which are the fundamentals of rational medical treatment of AD patients.

## Author Contributions

LS and PR developed the idea and concept for this article. LS wrote the manuscript with the contribution of JM, RP, PT, TL, LM, KB, and PR. LS, JM, and PT developed the figures. MH-A, AS, AM, and PR contributed to the interpretation of the results, figure development, and revision of the manuscript. All authors have made substantial intellectual contributions to this work and approved it for publication.

## Conflict of Interest

The authors declare that the research was conducted in the absence of any commercial or financial relationships that could be construed as a potential conflict of interest.
